# Risk-averse personalities have a systemically potentiated neuroendocrine stress axis: A multilevel experiment in *Parus major*

**DOI:** 10.1016/j.yhbeh.2017.05.011

**Published:** 2017-07

**Authors:** Alexander T. Baugh, Rebecca A. Senft, Marian Firke, Abigail Lauder, Julia Schroeder, Simone L. Meddle, Kees van Oers, Michaela Hau

**Affiliations:** a Department of Biology, 500 College Avenue, Swarthmore College, Swarthmore, PA 19081, USA; b Department of Life Sciences, Imperial College London, Silwood Park Campus, Buckhurst Road, SL5 7PY, Ascot, Berks, United Kingdom; c The Roslin Institute, The Royal (Dick) School of Veterinary Studies, The University of Edinburgh, Easter Bush, Midlothian EH25 9RG, United Kingdom; d Department of Animal Ecology, Netherlands Institute of Ecology (NIOO-KNAW), Droevendaalsesteeg 10, 6708 PB Wageningen, The Netherlands; e Evolutionary Physiology Group, Max Planck Institute for Ornithology, Eberhard-Gwinner-Str., 82319 Seewiesen, Germany; f Department of Biology, Universitätsstrasse 10, University of Konstanz, Konstanz, Germany

**Keywords:** ACTH, Behavioral syndromes, Corticosterone, Dexamethasone, Glucocorticoid receptor, HPA axis, Mineralocorticoid receptor, Negative feedback, Personality, Stress

## Abstract

Hormonal pleiotropy—the simultaneous influence of a single hormone on multiple traits—has been hypothesized as an important mechanism underlying personality, and circulating glucocorticoids are central to this idea. A major gap in our understanding is the neural basis for this link. Here we examine the stability and structure of behavioral, endocrine and neuroendocrine traits in a population of songbirds (*Parus major*). Upon identifying stable and covarying behavioral and endocrine traits, we test the hypothesis that risk-averse personalities exhibit a neuroendocrine stress axis that is systemically potentiated—characterized by stronger glucocorticoid reactivity and weaker negative feedback. We show high among-individual variation and covariation (i.e. personality) in risk-taking behaviors and demonstrate that four aspects of glucocorticoid physiology (baseline, stress response, negative feedback strength and adrenal sensitivity) are also repeatable and covary. Further, we establish that high expression of mineralocorticoid and low expression of glucocorticoid receptor in the brain are linked with systemically elevated plasma glucocorticoid levels and more risk-averse personalities. Our findings support the hypothesis that steroid hormones can exert pleiotropic effects that organize behavioral phenotypes and provide novel evidence that neuroendocrine factors robustly explain a large fraction of endocrine and personality variation.

## Highlights

• Hormonal pleiotropy is proposed as a mechanism for animal personality.
• We repeatedly measured behavior, CORT dynamics and neural MR/GR densities.
• MR/GR densities predict CORT dynamics and together predict behavioral variation.
• Birds with lower GR and more potentiated HPA dynamics were more risk averse.
• Stable and covarying behavioral and endocrine traits support hormonal pleiotropy.

## 1 Introduction

Upon exposure to a social or environmental challenge, individuals within a population often differ consistently in their behavioral response (reviewed in Réale et al., 2007, Bell et al., 2009, Dall et al., 2012). Moreover, single behaviors (e.g. aggressiveness) are often linked within an individual with other behaviors (e.g. exploration; reviewed in Groothuis and Carere, 2005). These consistent individual differences and trait correlations are the basis for the concept of animal personality (similar to ‘coping styles’, ‘behavioral syndromes’), which has now been demonstrated in a wide variety of species (van Oers and Naguib, 2013). This research highlights the constraints on behavioral flexibility, on the independent evolvability of traits, and suggests that the mechanisms that underlie one particular behavior might subserve other behaviors (Réale et al., 2007).

The hypothesis that hormones serve as mechanisms underpinning animal personality has been the subject of growing interest (Williams, 2008, Koolhaas et al., 2010). Glucocorticoids (hereafter CORT) are proposed to be key steroids involved in one of the major axes of personality: the shy-bold continuum (Øverli et al., 2007, Carere et al., 2010). In part, this hypothesis rests on the pleiotropic nature of steroids—these endocrine products circulate throughout the organism and bind to multiple receptor types across diverse tissues. Hence, a single hormone can simultaneously affect multiple targets, thereby precisely modulating the expression of several behaviors (Ketterson and Nolan, 1999).

As the end products of the hypothalamic-pituitary-adrenal (HPA) axis, CORT facilitate critical functions in vertebrates: coping metabolically with the fluctuating demands of normal life, such as day-night rhythmicity, locomotor activity and predictable daily and life-history events (Landys et al., 2006). Further, the HPA axis is essential for coping with unpredictable, acutely challenging events, such as exposure to unfamiliar environments or objects (Lendvai et al., 2011), inclement weather (Breuner and Hahn, 2003), predators (Cockrem and Silverin, 2002), but also sexual behaviors and social victory (Koolhaas et al., 2011). The regulation of the HPA axis consists of several components: First, low baseline concentrations fluctuate according to diel rhythms and metabolic demands and are known to promote feeding behavior (Dallman et al., 1993). Second, within a few minutes after an acute challenge is perceived, CORT (following an elevation of their upstream secretagogues such as adrenocorticotropic hormone, ACTH) becomes elevated and continues to rise in the blood until it reaches a peak, typically within 30–90 min (Baugh et al., 2013, Droste et al., 2011). At these stress-induced concentrations, CORT facilitates a metabolic shift from protein and fat synthesis towards gluconeogenesis by altering transcription in target cells (Gray et al., 1990, Hasselgren, 1999, Sapolsky et al., 2000, Oakley and Cidlowski, 2013). Third, negative feedback reduces circulating levels, allowing baseline concentrations to be re-achieved (Romero, 2004).

Regulation of circulating CORT concentrations is made possible by two intracellular receptors in the brain that bind CORT. The mineralocorticoid receptor (MR) has a high affinity and low capacity for CORT and is therefore thought to be principally active at baseline CORT concentrations (Romero, 2004, Landys et al., 2006). In contrast, the low affinity and high capacity glucocorticoid receptor (GR) exhibits increased binding at stress-induced concentrations (de Kloet, 1998, Funder, 1997) and is also thought to play a critical role in regulating negative feedback through binding to receptors located in the pituitary and hypothalamus, thereby inhibiting the secretagogues that lead to further elevations in CORT (de Kloet, 1991, Ronchi et al., 1998, Romero, 2004). Moreover, because of its upstream location in the HPA axis, receptor expression in the brain has the potential to explain intraspecific variation in stress physiology and behavior. Here we examine MR and GR expression in the hypothalamus and hippocampus, two brain regions known for their involvement in HPA regulation and roles in mediating behavior (Nelson, 2005). Higher GR expression in these regions, for example, might result in stronger negative feedback and thus a systemically less potentiated HPA axis (i.e. lower CORT at all post-stressor time-points).

Beyond single behaviors, the ways in which individuals respond hormonally to stressors may underlie several of the correlated behaviors that often characterize personality (Koolhaas et al., 2007). Further, if individuals vary consistently in functional aspects of the HPA axis—the circulating concentrations of glucocorticoids (CORT) and the expression patterns of receptors in behaviorally relevant tissues (e.g. nervous system)—this could give rise to variation in personality. Indeed, there is often remarkable intra-population variation in concentrations of baseline and stress-induced CORT (Hau et al., 2016). The fraction of this variation that represents among-individual variance has been studied in recent years and has yielded mixed results, reflecting in part the fact that only a subset of these studies used repeated measures designs (Baugh et al., 2014). However, understanding the endocrine basis of animal personality requires repeatedly characterizing behavioral, endocrine and neuroendocrine traits in the same individuals (reviewed in Ball and Balthazart, 2008)—a step that, to our knowledge, has not been undertaken until now.

In the present study we tested for the presence of among-individual variance in both behavioral traits and functional aspects of the HPA axis and then tested the hypothesis that variance in HPA axis function explains behavioral variance. Because environmental context can drive considerable acute variation in plasma glucocorticoids, we sought to control experimentally certain aspects of the environment—nutrition and exposure to conspecifics—but allowed physical aspects of the environment to vary naturally (e.g. weather). Using semi-natural enclosures, we studied wild-caught great tits (*Parus major*), a species that has been the subject of extensive investigation in animal personality (van Oers and Naguib, 2013) and, more recently, of intra-population variation in glucocorticoid physiology (Hau et al., 2016). We predicted that: (1) risk-taking behaviors expressed in the context of a foraging task will both vary at the among-individual level (i.e. exhibit repeatability) and covary at the among-individual level (i.e. exhibit syndromes); (2) four functional aspects of the HPA axis—baseline CORT, the stress response, negative feedback strength and adrenal sensitivity—will likewise vary and covary at the among-individual level; (3) the expression patterns of MR and GR in two regions of the brain that regulate the HPA axis (hippocampus and hypothalamus) will be correlated with HPA function, with higher GR expression predicted to strengthen negative feedback; and thus GR expression in these regions is predicted to correlate negatively with a systemically potentiated HPA axis (Romero, 2004); and (4) repeatable elements of the behavioral phenotype are correlated with repeatable elements of the endocrine phenotype; specifically, that birds with lower GR expression would express a consistently potentiated HPA stress axis and more risk-averse personalities.

## 2 Materials and methods

### 2.1 Animals

**Fig. 1:**
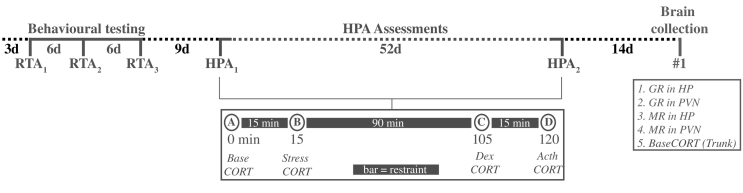
Experimental timeline. Birds were tested on a risk-taking assay on three occasions (RTA_1–3_) with 6-day intervals. Following a 9-day recovery period, they were sampled using a four component HPA assessment (A–D), once in August (HPA_1_) and again in November (HPA_2_) with a 52 day interval separating these two assessments. Following a 14-day recovery period, brains and trunk blood were harvested.

We used a repeated measures study design that included behavioral testing (N = 27; 15 females), plasma hormone assessment (N = 25; 13 females) and neural hormone receptor mRNA quantification (N = 25; 13 females; unequal sample sizes reflect the fact that two birds died of unknown causes between behavioral and hormonal assessments; Fig. 1). In 2009, we collected eggs from 14 nests (7 nests had clutch sizes of 1; 1 nest had a clutch size of 2; 6 nests had clutch sizes of 3) from an established nest box population (Westerheide, NL). Eggs were then distributed to unique and random wild foster parents to decouple nestling experience and relatedness among siblings. Because other maternal effects prior to hatching (e.g. yolk hormones) might influence the adult phenotype, we call this a ‘nest of origin’ effect (hereafter NestID) rather than strictly genetic relatedness. Ten days after hatching, fledglings were transported to the Netherlands Institute for Ecology (NIOO-KNAW, Heteren, NL) and hand-raised in captivity until nutritional independence.

In November 2010, the birds were transported by automobile to the Max Planck Institute for Ornithology-Radolfzell, where all experimental and laboratory work was conducted. After two weeks of quarantine, birds were housed singly in large outdoor aviaries (3 × 3 × 2 m high) in alternating male-female adjacencies (birds had audible but not visible contact). These captive conditions facilitated control of the social and nutritional environments—singly housed birds were fed an ad libitum diet and fresh water. Each aviary contained an elevated feeding platform, a nest box, hanging perches and live shrubs. Birds were acclimatized to these housing conditions for three months before testing began. We first characterized behavioral traits using three repeated samplings, and then characterized HPA axis function using two repeated measures sampling events, and lastly we sacrificed the birds to estimate the expression of hormone receptors in the brain (Fig. 1).

### 2.2 Behavioral testing

Twenty-seven birds were tested in a behavioral assay for object neophobia and risk-taking on three repeated occasions (RTA_1–3_; Fig. 1). Testing order was randomized with the exception that adjacent aviaries were never sampled on the same day and the two sexes were balanced each day. To ensure motivation and to habituate birds to feeding on the ground, each bird was restricted to three mealworms per day in a bowl centered on the floor of the aviary during a three-day window prior to testing. We tested a maximum of 7 birds per day during the morning (7:30–12:00). To habituate birds to the experimental set-up, we placed a camouflaged blind in front of each aviary at a distance of 3 m beginning 24 h prior to testing. The experimenter occupied the blind during the testing.

Our neophobia/risk-taking assessment was modified from a procedure previously validated as a measure of personality in this species (van Oers et al., 2005). Briefly, a small platform (30 cm^2^) containing live mealworms and a loaded mousetrap was introduced to the aviary floor and monofilament was used to trigger the trap from a distant observation blind (see Supplementary fig. 2). All birds alighted initially on the aviary floor and then hopped onto the front of the platform near the mealworm dish, at a distance of approximately 20 cm from the mousetrap. No birds were injured during the course of this assay.

We recorded (1) *Initial Latency*: time elapsed from the start of the trial until the bird approached the platform and retrieves a mealworm—because this is each bird's first exposure to the platform, we assume this latency reflects a low risk response to novelty (object neophobia); (2) *Reward Latency*: time elapsed since retrieval of the first worm and the return to the platform for a second mealworm (note: all birds flew to a shrub to consume each mealworm). Upon alighting on the platform for this second mealworm the experimenter triggered the trap, startling the bird (all birds flew away); and (3) *Startle Latency*: time elapsed between triggering the trap and the bird returning to the platform and retrieving a mealworm—we assume this latency reflects a high risk response. Trials in which birds did not return within the maximum trial duration (20 min) were not included in the repeatability, covariance or PCA analyses because assigning a constant value (e.g. 1200 s) would artificially inflate these estimates. Although, exclusion of these incomplete trials could under-represent the most risk-averse personalities, a pilot study conducted with wild-caught birds showed that extending this time window to longer durations (60 min) resulted in few additional latency data. The number of mealworms on the platform was counted following each trial to ensure that the expected number of worms was retrieved. This testing procedure was repeated three times per bird, with a three-day interval separating repeated trials. Upon completion of the third trial, each bird was measured for tarsus length, flattened wing cord length, body mass, and fat score (Cherry, 1982); body condition was estimated using the scaled mass index method (SMI; Pieg and Green, 2009), and completion of prebasic molt was confirmed.

### 2.3 HPA assessments

#### 2.3.1 Validation

In March 2012 we validated the HPA assessments following (Dickens et al., 2009a), including the pharmacological dosages, time courses, and the cross reactivity of the pharmacological reagents in the ELISA. We used wild-caught adult great tits—used only for this validation—from a nest box population in Radolfzell, Germany (N = 13) (Supplementary fig. 1).

#### 2.3.2 Blood collection

Twenty-five birds were tested for plasma glucocorticoid dynamics using a repeated measures HPA assessment (Fig. 1). The first assessment (HPA_1_) was conducted during a one-week period in late August following the third and final set of behavioral trials to facilitate analysis of hormone-behavior relationships. The second assessment (HPA_2_) was performed in November preceding the brain collection. Sampling was limited to 0800–1100. Samples were collected by puncturing the brachial vein and collecting the blood using a heparinized microcapillary tube. Birds were restrained in small cotton bags during the intervals between sampling time points.

Our method for assessing the HPA axis has been used previously in birds (Dickens et al., 2009a, Hau et al., 2015) and reptiles (Romero and Wikelski, 2010) to simultaneously quantify four aspects of HPA axis function: (1) Baseline CORT (*BaseCORT*): this first blood sample precedes the handling/restraint-induced stress response. *BaseCORT* was collected within 2 min following entry into the aviary to reduce contamination from the stress response (Baugh et al., 2013, Romero and Reed, 2005) and was followed by placing the bird in a small cotton restraint bag for 15 min. (2) Stress response (*StressCORT*): this second blood sample provides an estimate of the early stage of each bird's acute response to handling/restraint and was immediately followed by an intramuscular injection of dexamethasone (DEX; 1000 μg kg^− 1^; diluted to 50 μL in PBS), which stimulates strong negative feedback of the HPA axis, thereby down-regulating subsequent CORT secretion (Dickens et al., 2009a, Hau et al., 2015); this injection was followed by a 90-min restraint period. (3) Negative feedback strength (*DexCORT*): the CORT concentration here reflects the strength of negative feedback following the DEX injection (higher CORT here indicates *weaker* negative feedback); this was followed immediately by an intramuscular injection of adrenocorticotropic hormone (ACTH; Sigma #A6603; 100 IU kg^− 1^ diluted to 50 μL in PBS), followed by a 15-min restraint period. (4) Adrenal sensitivity (*ActhCORT*): finally, birds were bled a fourth time to estimate the capacity of the adrenal glands to produce CORT upon pharmacological stimulation of the HPA axis by the injected secretagogue. Birds were then immediately measured for biometrics, released into their aviary and monitored for health. Blood samples were kept on wet ice during sample collection and then centrifuged (1400 g for 10 min). The plasma fraction was frozen at —80C until all samples were assayed simultaneously.

#### 2.3.3 Enzyme immunoassay

In July 2013 we estimated plasma CORT concentrations using a commercial enzyme immunoassay kit (Enzo Life Sciences, Cat. No. ADI 900-097; Donkey anti-Sheep IgG). The details of our EIA procedure, including its validation, extraction, recoveries, technical repeatability and preparation of standards are reported in (Baugh et al., 2014, Ouyang et al., 2011). The intra- and inter-assay coefficients of variation (CV; 9 plates)—8.1% and 8.2%, respectively. The assay has a detection limit of 27 pg mL^− 1^. The cross-reactivity of the antiserum is 100% for corticosterone, 28.6% for deoxycorticosterone and 1.7% for progesterone.

#### 2.3.4 Neural receptors quantification

Following a 14-day recovery from the second HPA assessment, birds (N = 25) were captured by hand net and decapitated. Trunk blood was collected and kept on wet ice while whole brains were dissected from the skulls and frozen in aluminum foil on dry ice and maintained at —80C until cryosectioning. The interval separating entry into the aviary and frozen tissue was < 3 min. We transported brains on dry ice to the Roslin Institute at the University of Edinburgh, mounted them on OCT (TissueTek) and sectioned them coronally at 15 μm onto polysine pretreated slides. Tissue was stored at —80C. Two slides from each animal (each with six sections) were used for radioactive in situ hybridization and a custom Python script and ImageJ were used for silver grain quantification in the paraventricular nucleus of the hypothalamus (PVN) and the hippocampus (HP)—two regions implicated in HPA axis regulation (Dickens et al., 2009b). Details of our methods are described in (Senft et al., 2016).

### 2.4 Statistical analyses

#### 2.4.1 Behavior: general

We used a repeated measures ANOVA to test for effects of repeat number and latency type, including covariates for sex, SMI, and fat score. This permitted us to evaluate whether birds became habituated to the novel object platform across repeated trials. Effect sizes (partial eta-squared and Cohen's d) were calculated for significant main effects, interactions and pair-wise comparisons.

#### 2.4.2 Among-individual variances (i.e. repeatabilities)

All repeatability and covariance analyses were performed in R 3.0.2. We estimated the within- and among-individual variance components using Bayesian general linear mixed models (GLMMs) with a Gaussian error distribution, and used the variance component estimates to calculate the repeatability of each behavioral and hormonal trait (log_10_ transformed and z-standardized). Our linear mixed models approach (Sokal and Rohlf, 1995, Nakagawa and Schielzeth, 2010) has important advantages over earlier ANOVA-based methods for repeatability estimation (intra-class correlation coefficients; Lessells and Boag, 1987), including the incorporation of environmental covariates and nested terms (i.e. adjusted repeatabilities), robustness to data heterogeneity (missing values, unbalanced designs), and the ability to estimate uncertainty around the repeatability estimate because variances are estimated directly. These models were constructed in MCMCglmm (Hadfield, 2010). We ran models without fixed effects (i.e. agreement repeatabilities), with individual identity as the sole random effect, and used inverse-Wishart priors. A second set of models were conducted to correct our estimates for nest of origin effects and determine whether the nest of origin explained some of the variation in traits. Therefore, we added NestID as a random effect to the models. We then repeated all the above models, this time accounting for fixed effects (i.e., adjusted repeatabilities), by adding variation in body condition (SMI, scalar) and the fat score as fixed covariates (Supplementary table 4 for adjusted repeatabilities), with a similar prior. Sex was not included as a term in any of the models because behavioral, endocrinological and quantitative genetics studies in *P. major* have shown no evidence for sex-dependent expression of exploratory behavior (Dingemanse et al., 2002, van Oers et al., 2004a, Carere et al., 2005), risk-taking behavior (van Oers et al., 2005), or HPA axis function (Stöwe et al., 2010, Baugh et al., 2014).

We used the variance component estimates to calculate effects of individual identity and nest of origin. We ran each model for 1,000,000 iterations, used default sampling, and we ran model diagnostics to confirm that the autocorrelation between subsequent stored iterations was not higher than 0.1. We report the repeatabilities, and variance components calculated as a ratio of the total variance, with 95% credible intervals (95CI).

#### 2.4.3 Covariances

We performed bivariate GLMMs to estimate within- and among-individual covariation. These models were constructed in MCMCglmm (Hadfield, 2010), where individual identity was fitted as random effect. Covariances in both the random effect and the residual were allowed to take on any value (for a similar analysis and sample size, see Araya-Ajoy and Dingemanse, 2016). We compared the Deviance Information Criterion (DIC, Spiegelhalter et al., 2002) of these models with one from a model where we fixed the covariance within individuals to zero. A difference of > 5 in DIC was considered statistically significant (Spiegelhalter et al., 2002). We ran these models with uninformative priors and ran each model for 2,000,000 iterations. Standard model diagnostics confirmed that autocorrelation among sampled iterations was low.

#### 2.4.4 Linking neural receptors, HPA dynamics and behavior

HPA_2_ was timed to precede brain collection (Fig. 1) to test the hypothesis that MR and GR expression in the HP and PVN predict HPA axis function. To do this we used the average MR and GR expression in the PVN and HP calculated across four coronal sections per bird (N = 25). We constructed general linear models to describe how neural receptor expression predicts CORT concentrations for the four HPA components. We also included two fixed variables in these models to represent body condition, SMI and furcular fat score, that have been shown to be correlated with CORT secretion (Wingfield et al., 1994).

**Fig. 2:**
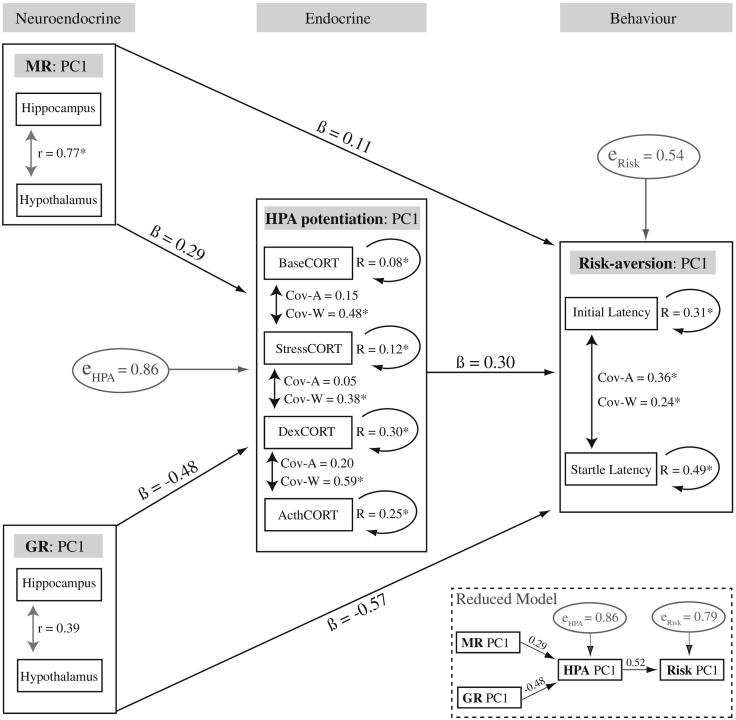
Relationships among traits depicted in a path model. For each level of organization (neuroendocrine: MR and GR expression; endocrine: HPA_1_ components; behavior: Initial and Startle Latencies), traits were reduced to the first principal component (PC1). For traits measured repeatedly per bird, agreement repeatabilities (R; subject as random factor) are indicated inside circular arrows. Estimates of covariance between traits within each category are indicated with bi-directional black arrows at the among- (Cov-A) and the within-individual levels (Cov-W). The phenotypic correlations (Pearson's r) for MR and GR across the two nuclei are indicated with bi-directional grey arrows. The path model yields beta coefficients (β) describing the direction and magnitude of effect of independent variables (GR, MR, HPA) on dependent variables (HPA, risk-taking). Error estimates for dependent variables in the path model are indicated in grey ellipses. The model indicates that higher MR expression in the hippocampus and hypothalamus and lower GR expression in these two brain areas predicts a more potentiated HPA axis (higher CORT) and more risk-averse personalities (higher latencies). Inset: the reduced model has two dropped paths. * denotes statistical significance (p < 0.05) for repeatability (R), covariance (Cov) and correlation (r) estimates.

To test the broader relationships among receptors, hormones and behavior—and in order to reduce family-wise error rates—we reduced the dimensionality for all three of these phenotypic categories using principal components analysis and then used path analyses (SPSS version 21) to test the strength and direction of relationships among phenotypic levels (Fig. 2). We tested two a priori models that minimized the number of paths: (1) Full model: MR and GR directly influence both HPA axis function and behavior and the HPA axis also directly influences behavior; and (2) Reduced model: MR and GR only indirectly influence behavior via the HPA axis. We calculated the fit of both models (1 – π(e_HPA_ ∗ e_Risk_)) and a summary statistic (Q = (1 − Fit_Full_) / (1 − Fit_Reduced_)) and then compared the significance of this quotient with a Chi-squared test of significance (W = −(N − d) ∗ log_e_(Q), where N = sample size and d = the number of dropped paths). Measurements from RTA_1_ and HPA_1_ were used and all datasets were log_10_-transformed and z-standardized prior to component extraction. For the behavioral data, we excluded trials in which the bird did not return to the platform within the maximum window of time (20 min) because assigning maximum values here would artificially inflate the eigenvalues (final N_path analysis_ = 14). All analyses extracted only one component (PC1) with eigenvalues > 1 (which explained 63–82% of the variance) and correlation matrices indicated that all pairwise correlation coefficients varied between 0.1 and 0.9 (Supplementary table 8). Therefore, we performed path analyses using PC1 for each trait category (Fig. 2). Residual error for these analyses did not deviate from Gaussian, visually or statistically (Shapiro-Wilk; all p > 0.20) and observed power for the omnibus path model was adequate (power = 0.83).

## 3 Results

### 3.1 Behavior: general

Sex (F_1_ = 0.674, p = 0.443), SMI (F_1_ = 0.044, p = 0.840) and fat score (F_3_ = 0.503, p = 0.694) did not explain significant variance in behavior. The repeated measures ANOVA therefore only included the within-subjects factors (latency type and repeat number; Supplementary fig. 2). Latencies were generally high for *Initial Latency* and *Startle Latency* (mean ± SD, sec: 347.8 ± 279.9; 283.3 ± 232.0, respectively)—indicating that this assay predictably elicited a neophobic and startle response, respectively—but low for *Reward Latency* (143.5 ± 163.8), suggesting that the first mealworm acted as a food reward. There was a significant main effect of latency type (F_2,26_ = 22.82, p < 0.001, partial η^2^ = 0.74). There was no main effect of repeat number (F_2,26_ = 1.54, p = 0.233), but there was an interaction between latency type and repeat number (F_4,52_ = 3.08, p = 0.024, partial η^2^ = 0.73) due to a marginal reduction in the *Initial Latency* between RTA_1_ and RTA_2_ (pairwise comparison: p = 0.01, Cohen's d = 0.92 (95CI = 0.19–1.64); Fig. 1). No other pairwise comparison was significant (Supplementary fig. 2), suggesting that the novelty of the platform diminishes over repeated exposures.

### 3.2 Behavioral variances

Repeatabilities for all three latencies were high (0.41–0.53; see Bell et al., 2009) and statistically significant (i.e. with 95CI that were not zero-bound; Table 1; Supplementary fig. 5(a–c)). NestID did not explain significant variance in the three behaviors (Table 1), suggesting that risk-taking phenotypes may not be strongly explained by relatedness or pre-hatching maternal effects (e.g. yolk hormones) or both. However, the statistical power to detect NestID effects was not particularly high because half of the nests did not have siblings, so we cannot exclude this possibility. Moreover, results stayed qualitatively the same when accounting for fat score and SMI (Supplementary table 4 for adjusted repeatabilities).

**Table 1:** Repeatability estimates and 95% confidence intervals for the three behavioral measures. Agreement (i.e. raw) repeatabilities are shown with and without the rand25 effect of NestID (i.e. siblings). Estimates for the variance explained by NestID and its 95% confidence interval are also shown for each behavior. Confidence intervals that are not zero-bound are considered statistically significant.

Behavioral trait		R	95CI	Nest ID	Nest ID 95CI
*Initial Latency*	Raw^a^	0.41	0.13–0.66	–	–
	Raw^b^	0.31	0.00–0.58	0.01	0.00–0.27
*Reward Latency*	Raw^a^	0.49	0.23–0.75	–	–
	Raw^b^	0.35	0.02–0.63	0.02	0.00–0.41
*Startle Latency*	Raw^a^	0.53	0.23–0.77	–	–
	Raw^b^	0.49	0.12–0.75	0.01	0.00–0.31

a Agreement repeatabilities (i.e. no fixed effects) with individual as sole random term.

b Agreement repeatabilities (i.e. no fixed effects) with individual and Nest ID fitted as random terms.

### 3.3 Behavioral covariances

**Fig. 3:**
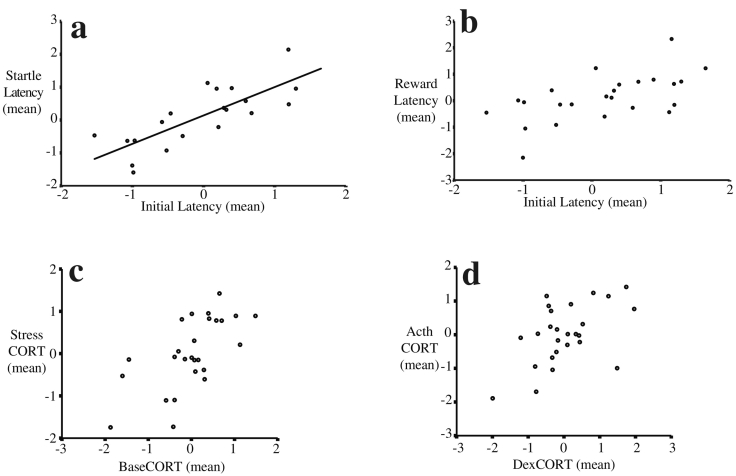
Graphical representations of the among-individual correlations for the three behavioral traits (a,b) and four HPA components (c,d). All values are log_10_-transformed and plotted as standardized (z) scores and best fit lines are linear regressions. Plots show the correlation between the average (per bird) values of the three behavioral trails (a,b) and two HPA assessments (c,d). A statistically significant positive correlation in (a) is graphical evidence of among-individual correlation (i.e. syndrome) between *Initial Latency* and *Startle Latency* (i.e. consistently neophilic birds are also consistently more risk-taking). The other trait pairs (b–d) were not statistically significant but positive trends here suggest the possibility of among-individual correlations.

There was a significant positive among-individual covariance between *Initial* and *Startle Latencies* (Table 3; Fig. 3a; Supplementary fig. 5(d–m)), demonstrating that birds that are chronically neophobic are chronically more risk-averse (i.e. personality). There was a similar trend between *Initial* and *Reward Latencies* (Table 3; Fig. 3b). The DIC of the model in which the covariance between *Initial Latency* and *Reward Latency* was fixed to zero did not fit the data better than a model in which the covariance was allowed to take on any value (∆ DIC = 13.6). This was also true for the model including covariances between *Initial Latency* and *Startle Latency* (∆ DIC = 23.8). In both models the covariance within-individuals was positive and statistically significant (95CI do not overlap zero), demonstrating that at a given moment in time a bird exhibiting more neophobic behavior will also predictably exhibit more risk-averse behavior (Table 3).

### 3.4 Hormones: general

Our validation study indicated that our drug dosages and timeline induced predictable variation in CORT concentrations, with low *BaseCORT*, moderately high *StressCORT*, low *DexCORT* and high *ActhCORT* (Supplementary fig. 1). In our experimental study we observed a main effect of HPA component (F_3,72_ = 186.8, p < 0.001, partial η^2^ = 0.97), again with low *BaseCORT* and *DexCORT*, and high *StressCORT* and *ActhCORT* (Supplementary fig. 3). There was also a main effect of season, with higher CORT concentrations in November (HPA_2_) compared to August (HPA_1_) (F_1,24_ = 24.29, p < 0.001, partial η^2^ = 0.51; Supplementary fig. 3). Lastly, there was an interaction effect between season and HPA component (F_3,72_ = 25.06, p < 0.001, partial η^2^ = 0.71), driven by weakened negative feedback (higher *DexCORT*) in November and a concomitant increase in *ActhCORT* (Supplementary fig. 3). The between-subject factors of sex (F_1,23_ = 3.46, p = 0.08), SMI (F_1,23_ = 2.5, p = 0.12), and fat score (F_1,23_ = 1.99, p = 0.17) did not significantly explain variance in the CORT variables and therefore were not included in the repeated measures ANOVAs.

### 3.5 Hormonal variances

One bird had a very low CORT concentration for the first *BaseCORT* sample. We ran models that included and excluded this statistical outlier, and the results did not differ qualitatively (direction and proportionality of estimates), thus we report the inclusive results. For all four traits, repeatabilities were statistically significant (i.e. 95CIs were not zero-bound). *DexCORT* and *ActhCORT* exhibited qualitatively higher repeatabilities than *BaseCORT* and *StressCORT,* but the 95CI did overlap so the estimates are not statistically significantly different from each other (Table 2). When accounting for NestID, the amount of variance explained by differences among individuals was qualitatively lower, and a larger part of the variance, especially for *BaseCORT*, was explained by NestID, but again, 95CIs overlapped (Table 2; Supplementary fig. 6(a–d)). Results stayed qualitatively the same when accounting for fat score and SMI (Supplementary table 4 for adjusted repeatabilities).

**Table 2:** Repeatability estimates and 95% confidence intervals for the four HPA components. Agreement (i.e. raw) repeatabilities are shown with or without the random effect of NestID (i.e. siblings). Estimates for the variance explained by NestID and its 95% confidence interval are also shown for each HPA trait. Confidence intervals that are not zero-bound are considered statistically significant. For repeatability estimates adjusted for body condition and fat score see Supplementary table 4. Repeatability estimates and 95% confidence intervals for the four HPA components. Agreement (i.e. raw) repeatabilities are shown with or without the random effect of NestID (i.e. siblings). Estimates for the variance explained by NestID and its 95% confidence interval are also shown for each HPA trait. Confidence intervals that are not zero-bound are considered statistically significant. For repeatability estimates adjusted for body condition and fat score see Supplementary table 4.

HPA trait		R	95CI	Nest ID	Nest ID 95CI
*BaseCORT*	Raw^a^	0.20	0.04–0.52	–	–
	Raw^b^	0.08	0.02–0.32	0.20	0.04–0.57
*StressCORT*	Raw^a^	0.31	0.08–0.59	–	–
	Raw^b^	0.12	0.03–0.43	0.12	0.03–0.48
*DexCORT*	Raw^a^	0.50	0.16–0.74	–	–
	Raw^b^	0.30	0.05–0.59	0.11	0.03–0.43
*ActhCORT*	Raw^a^	0.59	0.24–0.78	–	–
	Raw^b^	0.25	0.06–0.58	0.16	0.04–0.52

a Agreement repeatabilities (i.e. no fixed effects) with individual as sole random term.

b Agreement repeatabilities (i.e. no fixed effects) with individual and Nest ID fitted as random terms.

### 3.6 Hormonal covariances

The difference in DIC between the bivariate model of *BaseCORT* and the *StressCORT* where covariances were fixed to zero and where they were allowed to take on any value was large (∆ DIC = 27), thus we assumed that covariance modeling better explained the data. There were no statistically significant covariances at the among-individual level (Table 3; 95CI overlap zero), despite a trend for some trait pairs, including high *BaseCORT* being linked with a high *StressCORT* (Fig. 3c), and weak negative feedback (high *DexCORT*) linked to strong adrenal sensitivity (high *ActhCORT*) (Fig. 3d). The covariances within individuals for all trait pairs examined were statistically significant and positive (Table 3; Supplementary fig. 6(h,l,p)). Similarly, the model that included covariance between the *StressCORT* and *DexCORT* was statistically significant (∆ DIC = 19.5) and the covariance within individuals was statistically significant and positive, while the covariance among individuals was not different from zero (Table 3; Supplementary fig. 6(e–p)). Similarly, the covariances within individuals were statistically significant and positive for the pairs *DexCORT* versus *ActhCORT*, and *StressCORT* versus *ActhCORT*. Again, the differences in DICs confirmed that the models including covariance better explained the data (∆ DIC_Dex vs Acth_ = 26 and ∆ DIC_Stress vs Acth_ = 16).

**Table 3:** Estimates (and 95% confidence intervals) of within- and among-individual covariance for pairs of behavioral measures and pairs of HPA measures. Confidence intervals that do not overlap zero are considered statistically significant.

Trait pair	Cov among-individuals		Cov within-individuals	
	Estimate	95CI	Estimate	95CI
HPA				
*BaseCORT* vs *StressCORT*	0.15	− 0.08–0.45	0.48	0.21–0.81
*StressCORT* vs *DexCORT*	0.05	− 0.21–0.36	0.38	0.13–0.65
*StressCORT* vs *ActhCORT*	0.19	− 0.11–0.52	0.32	0.09–1.00
*DexCORT* vs *ActhCORT*	0.20	− 0.13–0.58	0.59	0.13–0.66
Behavior				
*Initial Latency* vs *Reward Latency*	0.05	− 0.35–0.48	0.23	0.06–0.44
*Initial Latency* vs *Startle Latency*	0.36	0.03–0.75	0.24	0.08–0.43

### 3.7 Linking neural receptors, HPA dynamics and behavior

Expression of GR was higher in the PVN than in the HP, and MR exhibited the opposite pattern. Expression of MR was positively correlated across the PVN and the HP and there was a trend for a positive correlation for GR across the two nuclei (Fig. 2 and Senft et al., 2016). In contrast, there were no correlations between the two receptor types (MR versus GR) for any combination of nuclei or PC (all p > 0.15).

Given the widespread covariance among trait items (e.g. *Initial* and *Startle Latencies*), the principal component analyses allowed us to reduce our dataset into four trait categories (MR phenotype; GR phenotype; HPA potentiation phenotype; risk-aversion phenotype; Fig. 2; see Supplementary table 8 for PCA details; Budaev, 2010) and minimize family-wise error rates. Using these trait categories, our path analyses indicated that higher MR and lower GR predict a more potentiated HPA axis (higher CORT) and more risk-averse (longer latencies) personalities (Fig. 2). Model fit was significantly higher for the full model (0.785) compared to the reduced model (0.539) with two dropped paths (χ^2^ = 9.12, df = 2, p < 0.05; Fig. 2), indicating support for a direct and an indirect (i.e., via HPA axis) influence of MR and GR on risk aversion. These results were robust to the structural details of the path model—the qualitative outcome did not change with inclusion/exclusion of items in principal components and the relationships among these higher-level trait dimensions mirrored patterns detected at lower levels of analysis. For example, variation in *ActhCORT* positively predicted startle latencies (R^2^ = 0.41, F_1,12_ = 8.36, p = 0.014); GR expression in the hippocampus negatively predicted startle latencies (R^2^ = 0.39, F_1,12_ = 7.76, p = 0.016); GR expression in the PVN negatively predicted *ActhCORT* (R^2^ = 0.24, F_1,23_ = 6.51, p = 0.019); and MR expression in the hippocampus positively predicted *StressCORT* (R^2^ = 0.20, F_1,23_ = 5.82, p = 0.024; see also Supplementary Table 7, Supplementary Table 8, Supplementary fig. 9).

## 4 Discussion

We found support for the hypothesis that avian personality is correlated with individual differences in HPA axis function. Our repeated measures design allowed us to partition variance in behavioral, endocrine and neuroendocrine traits in the same individuals under semi-natural conditions, providing an integrative picture of trait lability and interaction—to our knowledge this is the first such study to integrate across these levels of organization. The results support our prediction that individuals with consistently more potentiated HPA axes exhibit risk-averse personalities. These results help to unify findings from previous research examining the HPA axis and personality in vertebrates (Ellis et al., 2006, Koolhaas et al., 1999) including great tits (Hau et al., 2016) and provide an important test of the assumption that pharmacological challenges provide a window into upstream neural receptor phenotypes.

### 4.1 Personality

Risk-taking behaviors were repeatable—approximately 30–50% of the variation in each can be attributed to individual differences, a relatively high fraction for behavioral traits (Bell et al., 2009). And in contrast to the HPA components, nest of origin explained only a small fraction of the behavioral variation. This finding extends previous work that indicates that these types of risk-taking behaviors are components of a more general personality suite that includes exploration and boldness (van Oers et al., 2004b, van Oers et al., 2004c, van Oers et al., 2005, Hall et al., 2015). Moreover, these behaviors are phenotypically correlated, owing to the joint contributions of positive among- and within-individual correlations. The within-individual correlation implies that either dynamic internal (e.g., circadian state) or external variables (e.g., temperature) or both varied across observations of the same individual and that these variables modulated the expression of both traits simultaneously (see Baugh et al., 2014). Given that we controlled the social and nutritional environments, this within-individual correlation further indicates that these two behaviors are codependent on a variety of influences beyond nutritional state and social context (see Drosmann et al., 2014). This within-individual correlation could emerge as a consequence of ultradian cycles (Droste et al., 2011); for example, baseline CORT and stress-induced CORT will both be higher following the periodic and pulsatile release of ACTH. The among-individual correlation between these two behaviors, however, provides evidence for their joint contribution to personality and is only possible given the among-individual variance (i.e. repeatability) of both behaviors (Baugh et al., 2014). This correlation means that birds that are on average more neophobic are also on average more risk-averse, and vice versa. This is consistent with the finding in this species that risk-taking behavior is genetically linked with general aspects of the shy-bold continuum, including spatial and object neophobia (van Oers et al., 2005).

### 4.2 HPA function

Our HPA assessments yielded the predicted results (i.e. low *BaseCORT*, stress-induced increases, decrease after DEX- and increase following ACTH-injection). This is similar to what has been documented in some other species (Romero and Wikelski, 2010, Schmidt et al., 2012, MacDougall-Shackleton et al., 2013, Hau et al., 2015), but differs from the lack of ACTH sensitivity reported in chukar partridge (*Alectoris chuckar*; Dickens et al., 2009a). The higher CORT in November compared August suggests a seasonal pattern in DEX sensitivity, with weaker sensitivity in November. There was also a seasonal increase in *ActhCORT*, but this is likely due to the positive correlation between *DexCORT* and *ActhCORT*. Although our birds had recently completed prebasic molt at the time of the August assessment, the enduring physiological consequences of a recent molt or other seasonally variable inputs to the HPA axis might have downregulated axis sensitivity (Romero, 2006). Despite this seasonality, all four HPA measures exhibited significant repeatability.

Baseline CORT exhibited the lowest repeatability, consistent with previous studies in great tits (Baugh et al., 2014) and other species (Hau et al., 2015, Ouyang et al., 2011). The other three HPA components contained moderate (*StressCORT*) to high (*DexCORT*, *ActhCORT*) amounts of repeatability, consistent with the only other report that has estimated repeatabilities in a subset of these traits (Hau et al., 2015). The repeatabilities of *DexCORT* and *ActhCORT* might be interesting to examine in future functional studies. Variation in negative feedback, for example, has been shown to correlate with nutritional state and mortality in Galapagos marine iguanas (*Amblyrhynchus cristatus*; Romero and Wikelski, 2010).

We also demonstrated that there are positive phenotypic correlations between several of these HPA components. Most of the covariance between traits is at the within-individual level. In other words, this suggests that a bird with high baseline CORT at a particular moment will have high stress-induced CORT minutes later; but that same bird a month later might have moderate initial CORT and moderate stress-induced CORT. This hypothetical bird is a relatively high-CORT individual (i.e. there is repeatability in both components), but dynamic variables, not stable individual differences, are driving the correlation between the HPA traits (Baugh et al., 2014). The interdependence of these HPA measures has implications for coping with repeated stressors; for example, a positive trend between *DexCORT* and *ActhCORT* suggests that birds with stronger negative feedback are less able to mount a strong secondary stress response, owing perhaps to a refractory state. To our knowledge, these are the first estimates of combined phenotypic, within- and among-individual covariances in these HPA axis traits. Given trends indicating among-individual correlations between *BaseCORT* and *StressCORT* and between *DexCORT* and *ActhCORT*, we suggest future research examine this question further. However, because *BaseCORT* and *StressCORT* concentrations are likely regulated by separate receptor populations, thereby potentially decoupling them, we would not predict strong correlations between these measures (de Kloet et al., 1993, Romero, 2004).

### 4.3 Linking neural receptors, HPA dynamics and behavior

By estimating the expression of MR and GR in two brain regions known to regulate the HPA axis—the PVN and hippocampus—we provide support for the hypothesis that functional aspects of HPA axis dynamics can be predicted on the basis of receptor expression. Overall, we showed that higher MR levels predicted a more potentiated HPA axis (higher CORT) and, more importantly, higher GR levels predicted a less potentiated axis. Previously we showed that the expression of hippocampal MR is positively correlated with the expression of MR in the PVN, and a similar trend was observed for GR across these two nuclei (Senft et al., 2016). This within-individual correlation across nuclei is similar to what has been shown across diverse tissues in songbirds (Lattin et al., 2015) and suggests a neuroendocrine suite that constrains the flexibility of HPA axis function, which might explain individual differences in the HPA components measured in this study. Future research should also quantify receptor protein expression, as transcript and protein levels have been shown to be uncorrelated in house sparrow (*Passer domesticus*) brains (Medina et al., 2013), and transcript expression might yield different results. For example, in vivo studies in rodents have demonstrated that GR under-expression, which leads to reduced negative feedback (i.e. more potentiated HPA axis) can be compensated for by MR over-expression (Harris et al., 2013), suggesting that higher MR densities in rodent brains might be associated with increased negative feedback (i.e. less potentiated HPA axis). The classic model that MR strictly controls basal HPA drive whereas GR controls negative feedback is being revisited (Kolber et al., 2009), and there is empirical evidence that it is the ratio of MR and GR that might be critical to understanding HPA axis function (Harris et al., 2013). Lastly, because MR and GR are widely distributed throughout the brain, including in *P. major* (Senft et al., 2016), links between receptor expression and behavior need to be more thoroughly explored.

We found support for the hypothesis that variation in the neuroendocrine stress axis is correlated with personality. Specifically, more neophobic and risk-averse birds exhibited a more potentiated stress axis. This finding was confirmed with data reduction methods showing that the three behavioral measures were largely explained by a single principal component, and likewise for the four HPA components. PC1 for the behavioral measures might represent behavioral inhibition under conditions of risk and PC1 for the HPA components might represent endocrine potentiation. These two principal components were significantly and positively correlated: birds exhibiting more risk-averse personalities also exhibited more potentiated HPA axes. It is remarkable that the expression patterns of two receptors in the brain can explain such a large amount of variation in HPA axis function and animal personality. We agree with Ball and Balthazart (2008) that identifying functional interrelationships between behavior and hormones at the individual level, which has occasionally failed (Crews, 1998, Adkins-Regan, 2005), is greatly facilitated by inclusion of target tissue variables such as neural receptor expression. We would further propose that understanding the behavior and hormonal sides of the equation are greatly enhanced by multilevel approaches, including the characterization of syndromes. Lastly, because recent work has demonstrated links between immune function and risk-taking behavior (Jacques-Hamilton et al., 2017) and because glucocorticoids are known to suppress the immune system, it will be important for future work to integrate among all three of these phenotypic categories in wild animals.

These patterns expand on earlier studies in this species; we previously demonstrated a genetic correlation between spatial/object neophobia and glucocorticoid reactivity, with shy selection line birds exhibiting stronger stress responses (Baugh et al., 2012). Likewise, in a study of wild birds we showed that slower explorers exhibited faster and more enduring glucocorticoid responses (Baugh et al., 2013). The present study, which used pharmacological challenges in addition to the more conventional stress series, confirms that the more enduring stress response in more risk-averse personalities is due to weaker negative feedback per se. These results are generally consistent with studies of stress and personality in other vertebrates (Baugh et al., 2012). What is still unclear, however, is the directionality and causality of these hormone-behavior relationships (Koolhaas et al., 2010). Further study is needed to examine the developmental-organizational programming of HPA and behavior phenotypes (Schmidt et al., 2013, Marasco et al., 2016) and new techniques are needed to acutely manipulate endocrine function in a physiologically relevant manner.

## 5 Conclusions

Our multilevel and integrative approach—from neural receptor expression to plasma hormone dynamics and behavior—provides new insight into the network of trait (co)variances that are associated with animal personality. Our results support the hypothesis that two hormone receptors in the brain play an integral role in HPA axis function, which is in turn associated with predictable variation in personality. Further, our findings support the hypothesis that pleiotropic effects of steroid hormones can act as proximate mechanisms that integrate behavioral traits into personality suites. Overall, these results unify earlier research documenting the relationship between endocrine stress reactivity and the shy-bold continuum in songbirds. Lastly, if the widespread trait covariances shown here are the consequences of genetic correlations, as has been demonstrated previously for specific HPA-behavior trait pairs (Baugh et al., 2012), this would imply that selection targeting any one of these levels might affect the evolution of suites of concerted traits (Ketterson and Nolan, 1999, McGlothlin and Ketterson, 2008).

The following are the supplementary data related to this article.

Supplementary Fig. 1 a. HPA assessment validation. In March 2012 we collected 13 adult *Parus major* (7 females, 6 males) from a nestbox population near Radolfzell in Baden-Wuerttemberg, Germany (permit 35–9185.81/G-10/76 by District administration Freiburg Department of Agriculture, Rural areas, Veterinary and Food Administration, Baden-Wuerttemberg, Germany at the Max Planck Inst. Ornithology, Radolfzell to MH and ATB). Birds were housed singly in non-adjacent aviaries described in the Methods and allowed to acclimatize for two weeks. Blood sampling was carried out between 0730 and 1200 on 30-March-2012. All blood samples were collected by puncturing the alar vein with a 27 gauge syringe and collecting the upwelling blood (ca. 30 μL) using a heparinized microcapillary tube. The HPA assessment involved the following steps: (1) we quickly entered each aviary and hand-netted birds and rapidly (< 3 min from entry) collected an initial blood sample (*BaseCORT*) and placed each bird in a small cloth restraint bag for 15 min; (2) next, birds were quickly removed from the cloth bag and bled again (*StressCORT*) followed immediately by an intramuscular injection of dexamethasone (DEX; 1000 μg kg^− 1^ diluted to 50 μL in PBS) or vehicle (PBS) and then placed in a cloth bag for 90 min; (3) next, birds were removed from the cloth bag and bled again (*DexCORT*) followed by an intramuscular injection of ACTH (Sigma #A6603; 100 IU kg^− 1^ diluted to 50 μL in PBS) or vehicle (50 μL PBS) and returned to the restraint bag for 15 min; (4) finally, a fourth blood sample was collected (ActhCORT) and the bird was released into its aviary. Blood samples were kept on wet ice during sample collection and then immediately centrifuged (1400 g for 10 min) and the plasma fraction was frozen at —80 °C until assayed. We used commercial enzyme immunoassay kits ((Enzo Life Sciences, Cat. No. ADI 900-097; Donkey anti-Sheep IgG). See Methods for details on the protocol (note: the one exception to the Methods is that the mean CORT values are corrected assuming a 90% recovery). Our intra- and inter-assay (2 plates) coefficients of variation for this validation were 13.21% and 14.02%, respectively. The above graph illustrates the efficacy of our dosages and timeline: initial CORT levels are low and similar to concentrations in field collected birds (see Baugh et al., 2014); a 15-min restraint period is sufficient to unanimously induce a strong natural stress response; our DEX dosage effectively induced strong negative feedback at 90 min post injection in all 9 birds, whereas vehicle injected animals retained elevated stress induced CORT concentrations; our ACTH dosage effectively re-instates a strong stress response compared to PBS and it exceeds the natural stress response (pharmacological sensitivity is being estimated). We determined that neither ACTH nor DEX cross-reacted appreciably with the EIA by spiking stripped great tit plasma with each drug at the average injected concentrations (e.g. 16 μg DEX). Upon correcting for the physiological drug concentrations (songbirds are approximately 8% blood by body mass, thus an average great tit (16 g) is approximately 1.3 mL whole blood), by linear transformation, the levels of CORT in the ACTH (N = 2) and DEX (N = 4) spiked wells would be undetectable. The uncorrected estimates–i.e. CORT concentrations detected in wells spiked at 12000% the concentrations of the experienced drugs–were 1.2–1.7 ng mL^− 1^ for DEX and 0.48 ng mL^− 1^ for ACTH. Although this assumes a linear relationship between drug concentration and antibody cross reactivity, it also assumes zero clearance of the drug during the 15 or 90 min period after drug injection before the post injection blood samples are actually collected. Thus, given what would be a massive dilution in blood and the likely non-zero clearance, these results suggest no appreciable cross-reactivity of DEX and ACTH in the Enzo CORT EIA. Notice that the second PBS injection on the Full Control treatment results in an elevation equivalent to the ACTH injection group (120 min). This is likely due to a positive within-individual correlation, i.e. because these PBS birds had relatively high CORT at the 105 min timepoint (because PBS failed to induce negative feedback), the second injection (another stressor) facilitated a high secondary stress response. Supplementary fig. 1b (inset box). To illustrate the effect of treatment, all three treatment groups are collapsed for the *BaseCORT* and *StressCORT* time points since they have a shared treatment experience up until that time point. Supplementary Fig. 1

Supplementary Fig. 2 Descriptive statistics for behavioral latencies. Light grey bars represent the Initial Latencies (neophobia response); medium grey bars represent the Reward Latencies; dark grey bars represent the Startle Latencies (risk-taking response). Values in parentheses indicate number of completed trials. There was a significant main effect of latency type (mean ± SD, sec: *Initial Latencies*: 347.8 ± 279.9; *Reward Latencies*: 143.5 ± 163.8; *Startle Latencies*: 283.3 ± 232.0; F_2_ = 22.82, p = 2 × 10^− 6^). There was no main effect of repeat number (F_2_ = 1.54, p = 0.233), suggesting that there was generally no habituation/sensitization effect of repeated measurements. There was, however, an interaction between latency type and repeat number (F_4_ = 3.08, p = 0.024), which was due to a reduction in the Initial Latencies between the first and second repeated trials (pairwise comparison: p = 0.01; no other pairwise comparison was significant), perhaps suggesting that *Initial Latency* measurements are sensitive to repeated measures wherein birds become habituated as the novelty of the platform declines. Supplementary Fig. 2

Supplementary Fig. 3 Repeated HPA assessments #1 (August 2012) and #2 (November 2012). Corticosterone concentrations were lower in August (main effect of season: F_1,24_ = 24.29, p < 5 × 10^− 6^) and there was a main effect of HPA component (F_3,72_ = 186.8, p = 1 × 10^− 7^). Lastly, there was an interaction effect between season and HPA component (F_3,72_ = 25.06, p < 1 × 10^− 7^), driven by weakened negative feedback (higher *DexCORT*) in November and a concomitant increase in adrenal sensitivity (higher *ActhCORT*). These results mirror the validation study (see Supplementary fig. 1). Supplementary Fig. 3

Supplementary Table 4 Adjusted repeatability estimates for the behavioral measures and the four HPA components. ^§^ Adjusted repeatabilities have SMI and fat score fitted as fixed effects (covariates) and individual as random effect for the R column and individual and nest of origin for the Nest ID column. ^††^ Adjusted repeatabilities have SMI fitted as a fixed effect (covariate) and fat score fitted as a fixed effect (factor). Supplementary Table 4

Supplementary Fig. 5 Graphical representations of variance and covariance components for the three behavioral traits (*Initial Latency*, *Reward Latency*, *Startle Latency*). All values are log_10_-transformed and plotted as standardized (z) scores and best fit lines are linear regressions. Plots a–c provide a partial (the first two of three repeated trials) illustration of the repeatability of each behavior. Positive correlations graphically indicate repeatable traits. See Table 1 for the repeatability estimates. Plots d–f illustrate the phenotypic correlation between Initial and Reward Latencies (i.e. the correlation between the traits during each sampling period). The positive correlations here indicate that there exists either a within- or an among-individual correlation (or both jointly) between these two traits. Plot g shows the correlation between the average (per bird) values of Initial and Reward Latencies across all three repeated trials. A positive correlation here is graphical evidence of an among-individual correlation (i.e. syndrome) between these two traits. Plot h depicts the correlation between the deviation from the average (per bird) values of Initial and Startle Latencies across all three repeated trials (i.e. each bird has three points on this plot). A positive correlation here is graphical evidence of a within-individual correlation (i.e. plasticity) between these two traits. Plots i–m illustrate the same relationships for Initial vs Startle Latencies. All graphs with linear regression lines demonstrated statistical signficance (see Table 1 for repeatability estimates and Table 3 for the within- and among-individual covariance estimates). See Baugh et al. (2014) for a complete description of these graphical methods. Supplementary Fig. 5

Supplementary Fig. 6 Graphical representations of variance and covariance components for the four HPA components (*BaseCORT*, *StressCORT*, *DexCORT*, *ActhCORT*). All values are log_10_-transformed and plotted as standardized (z) scores and best fit lines are linear regressions. Plots a–d provide an illustration of the repeatability of each trait. Positive correlations graphically indicate repeatable traits. See Table 2 for the repeatability estimates. Plots e–f illustrate the phenotypic correlation between *BaseCORT* and *StressCORT* (i.e. the correlation between the traits during each sampling period). The positive correlations here indicate that there exists either a within- or an among-individual correlation (or both jointly) between these two traits. Plot g shows the correlation between the average (per bird) values of *BaseCORT* and *StressCORT* across both repeated HPA assessments. A positive correlation here is graphical evidence of an among-individual correlation (i.e. syndrome) between these two traits. Plot h depicts the correlation between the deviation from the average (per bird) values of *BaseCORT* and *StressCORT* across both repeated HPA assessments (i.e. each bird has two points on this plot). A positive correlation here is graphical evidence of a within-individual correlation (i.e. plasticity) between these two traits. See Table 3 for the within- and among-individual covariance estimates. Plots i–l illustrate the same relationships for StressCORT vs DexCORT, and plots m–p do so for *DexCORT* vs *ActhCORT*. All graphs with linear regression lines demonstrated statistical signficance (see Table 2 for repeatability estimates and Table 3 for the within- and among-individual covariance estimates). See Baugh et al. (2014) for a complete description of these graphical methods. Supplementary Fig. 6

Supplementary Table 7 a. General linear models predicting the second HPA assessment CORT secretion using log_10_ transformed MR and GR expression (in the PVN and HP) and log_10_ transformed CORT concentrations, with SMI as a covariate and fat score as a cofactor. Significant models (p < 0.05) are shown in white squares and non-significant models in grey squares. Bold text indicates the receptor expression was significant (p < 0.05) in predicting CORT concentrations in that model. Akaike Information Criteria corrected for small sample size (AICc; N = 25) are given for each model to evaluate fit, with smaller values indicating a better fit. Omnibus (“model”) statistics are reported in addition to statistics for the specific effect of receptor expression. Results were qualitatively identical when we applied hierarchical linear modeling techniques with NestID as a random factor.
b. General linear models without fixed effects predicting the HPA_2_ (November samples) components using log_10_ transformed MR and GR expression (in the PVN and HP) and log_10_ transformed CORT concentrations. Akaike Information Criteria corrected for small sample size (AICc; N = 25) are given for each model to evaluate fit, with smaller values indicating a better fit. Significant models (p < 0.05) are shown in white squares and nonsignificant models in grey squares. Results were qualitatively identical when we applied standard least squares regression techniques. Supplementary Table 7

Supplementary Table 8 Component loadings and variance explained for items in the principal components analyses. All data were log_10_ transformed and z-standardized prior to PCA analysis. Supplementary Table 8

Supplementary Fig. 9 Correlations between the natural stress response (*StressCORT*) and Initial Latencies (a) and negative feedback (*DexCORT*) and Startle Latencies (b) from HPA_1_ assessment and RTA_1_, respectively. Larger *StressCORT* values indicate a stronger stress response and larger *DexCORT* values indicate weaker negative feedback strength (i.e. higher CORT concentrations following dexamethasone challenge). Trials in which birds did not approach the platform within the 20 min criterion are included; exclusion of these birds do not qualitatively change these linear relationships. Supplementary Fig. 9

## Ethics

Our protocols were approved under permit 35-9185.81/G-10/76 by District Administration Freiburg Department of Agriculture, Rural Areas, Veterinary and Food Administration, Baden-Wuerttemberg, Germany at the Max Planck Institute for Ornithology, Radolfzell, Germany (MH, ATB) and permit NIOO.10-06 of the Royal Dutch Academy of Sciences (DEC-KNAW) to KVO.

## Competing interest

We have no competing interests.

## Funding

This work was supported by the Alexander von Humboldt Foundation grant number 1141248 (ATB), the Max Planck Society (MH), The Netherlands Institute of Ecology (NIOO-KNAW; KVO), The Roslin Institute at the University of Edinburgh (SLM), HHMI and the Biology Department at Swarthmore College (ATB, RAS, MF, AL).
