# Supplementary material for: Risk-averse personalities have a systemically potentiated neuroendocrine stress axis: A multilevel experiment in Parus major
Source: Horm Behav. 2017 Jul;93:99–108. doi: 10.1016/j.yhbeh.2017.05.011 (PMC5552616; doi:10.1016/j.yhbeh.2017.05.011)
Supplement: Supplementary Fig. 1 — a. HPA assessment validation. In March 2012 we collected 13 adult Parus major (7 females, 6 males) from a nestbox population near Radolfzell in Baden-Wuerttemberg, Germany (permit 35–9185.81/G-10/76 by District administration Freiburg Department of Agriculture, Rural areas, Veterinary and Food Administration, Baden-Wuerttemberg, Germany at the Max Planck Inst. Ornithology, Radolfzell to MH and ATB). Birds were housed singly in non-adjacent aviaries described in the Methods and allowed to acclimatize for two weeks. Blood sampling was carried out between 0730 and 1200 on 30-March-2012. All blood samples were collected by puncturing the alar vein with a 27 gauge syringe and collecting the upwelling blood (ca. 30 μL) using a heparinized microcapillary tube. The HPA assessment involved the following steps: (1) we quickly entered each aviary and hand-netted birds and rapidly (< 3 min from entry) collected an initial blood sample (BaseCORT) and placed each bird in a small cloth restraint bag for 15 min; (2) next, birds were quickly removed from the cloth bag and bled again (StressCORT) followed immediately by an intramuscular injection of dexamethasone (DEX; 1000 μg kg− 1 diluted to 50 μL in PBS) or vehicle (PBS) and then placed in a cloth bag for 90 min; (3) next, birds were removed from the cloth bag and bled again (DexCORT) followed by an intramuscular injection of ACTH (Sigma #A6603; 100 IU kg− 1 diluted to 50 μL in PBS) or vehicle (50 μL PBS) and returned to the restraint bag for 15 min; (4) finally, a fourth blood sample was collected (ActhCORT) and the bird was released into its aviary. Blood samples were kept on wet ice during sample collection and then immediately centrifuged (1400 g for 10 min) and the plasma fraction was frozen at —80 °C until assayed. We used commercial enzyme immunoassay kits ((Enzo Life Sciences, Cat. No. ADI 900-097; Donkey anti-Sheep IgG). See Methods for details on the protocol (note: the one exception to the Methods is that the mean CORT [file mmc1.pdf]

S1

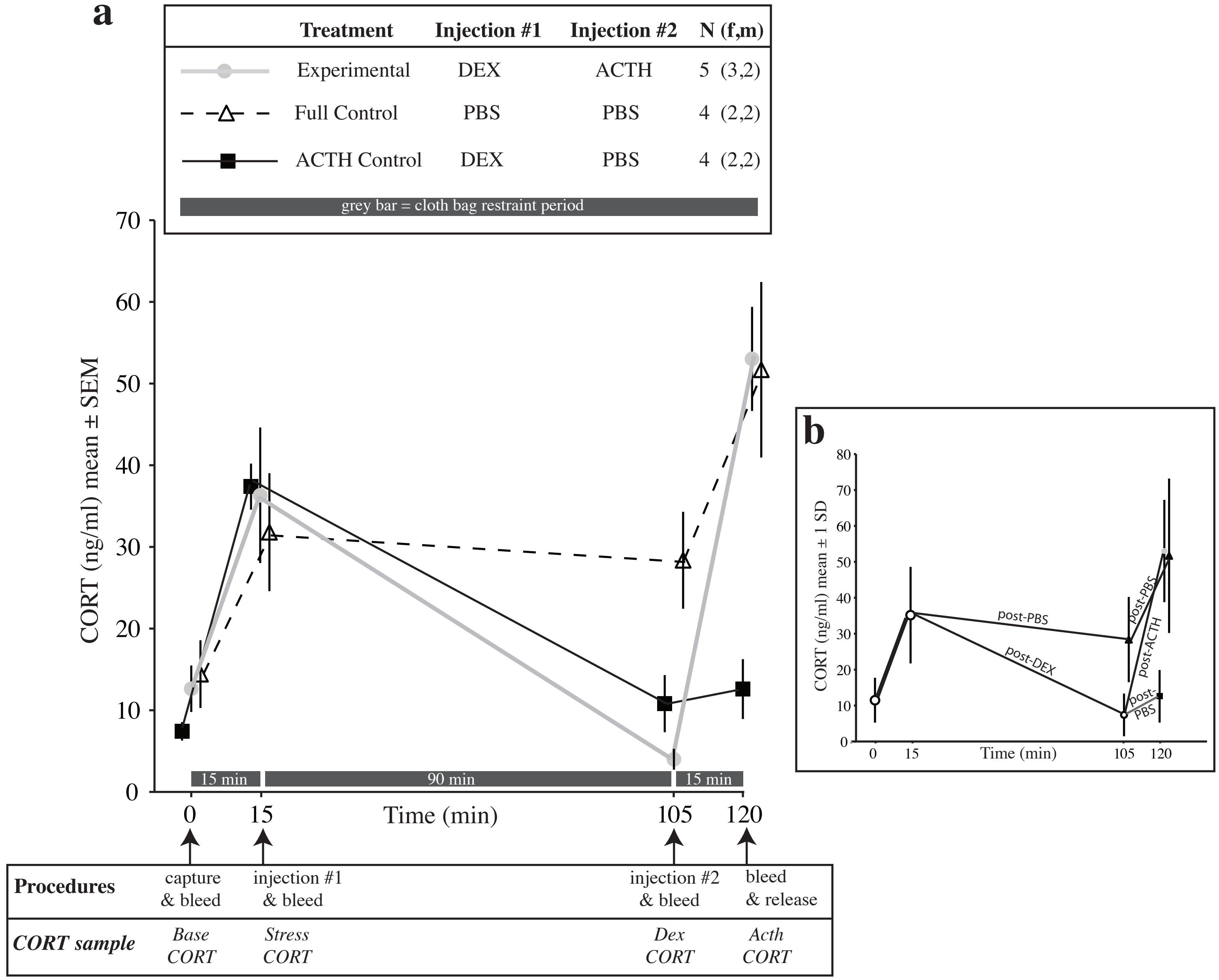

S1a. HPA Assessment Validation. In March 2012 we collected 13 adult *Parus major* (7 females, 6 males) from a nestbox population near Radolfzell in Baden-Wuerttemberg, Germany (permit 35–9185.81/G-10/76 by District administration Freiburg Department of Agriculture, Rural areas, Veterinary and Food Administration, Baden-Wuerttemberg, Germany at the Max Planck Inst. Ornithology, Radolfzell to MH and ATB). Birds were housed singly in non-adjacent aviaries described in the Methods and allowed to acclimitize for two weeks. Blood sampling was carried out between 0730-1200 on 30-March-2012. All blood samples were collected by puncturing the alar vein with a 27 gauge syringe and collecting the upwelling blood (ca. 30 μL) using a heparinized microcapillary tube. The HPA assessment involved the following steps: (1) we quickly entered each aviary and hand-netted birds and rapidly (<3 min from entry) collected an initial blood sample (*BaseCORT*) and placed each bird in a small cloth restraint bag for 15 min; (2) next, birds were quickly removed from the cloth bag and bled again (*StressCORT*) followed immediatley by an intramuscular injection of dexamethasone (DEX; 1000 μg kg^{-1} diluted to 50 μL in PBS) or vehicle (PBS) and then placed in a cloth bag for 90 min; (3) next, birds were removed from the cloth bag and bled again (*DexCORT*) followed by an intramuscular injection of ACTH (Sigma #A6603; 100 IU kg^{-1} diluted to 50 μL in PBS) or vehicle (50 μL PBS) and returned to the restraint bag for 15 min; (4) finally, a fourth blood sample was collected (ActhCORT) and the bird was released into its aviary. Blood samples were kept on wet ice during sample collection and then immediatley centrifuged (1400 g for 10 min) and the plasma fraction was frozen at -80 C until assayed. We used commerical enzyme immunoassay kits ((Enzo Life Sciences, Cat. No. ADI 900-097; Donkey anti-Sheep IgG). See Methods for details on the protocol (note: the one exception tothe Methods is that the mean CORT values are corrected assuming a 90% recovery). Our intra- and inter-assay (2 plates) coefficients of variation for this validation were 13.21% and 14.02%, respectively. The above graph illustrates the efficacy of our dosages and timeline: initial CORT levels are low and similar to concentrations in field collected birds (see Baugh et al., 2014); a 15-min restraint period is sufficient to unanimously induce a strong natural stress response; our DEX dosage effectively induced strong negative feedback at 90 min post injection in all 9 birds, whereas vehicle injected animals retained elevated stress induced CORT concentrations; our ACTH dosage effectively re-instates a strong stress response compared to PBS and it exceeds the natural stress response (pharmacological sensitivity is being estimated). We determined that neither ACTH nor DEX cross-reacted appreciably with the EIA by spiking stripped great tit plasma with each drug at the average injected concentrations (e.g. 16 ug DEX). Upon correcting for the physiological drug concentrations (songbirds are approximatley 8% blood by body mass, thus an average great tit (16 g) is approximately 1.3 mL whole blood), by linear transormation, the levels of CORT in the ACTH (N=2) and DEX (N=4) spiked wells would be undetectable. The uncorrected estimates--i.e. CORT concentrations detected in wells spiked at 12000% the concentrations of the experienced drugs--were 1.2-1.7 ng mL^{-1} for DEX and 0.48 ng mL^{-1} for ACTH. Although this assumes a linear relationship between drug concentration and antibody cross reactivity, it also assumes zero clearance of the drug during the 15 or 90 min period after drug injection before the post injection blood samples are actually collected. Thus, given what would be a massive dilution in blood and the likely non-zero clearance, these results suggest no appreciable cross-reactivity of DEX and ACTH in the Enzo CORT EIA. Notice that the second PBS injection on the Full Control treatment results in an elevation equivalent to the ACTH injection group (120 min). This is likely due to a positive within-individual correlation, i.e. because these PBS birds had relatively high CORT at the 105 min timepoint (because PBS failed to induce negative feedback), the second injection (another stressor) faciliated a high secondary stress response. **S1b (inset box).** To illustrate the effect of treatment, all three treatment groups are collapsed for the BaseCORT and StressCORT time points since they have a shared treatment experience up until that time point.
