# Supplementary material for: Risk-averse personalities have a systemically potentiated neuroendocrine stress axis: A multilevel experiment in Parus major
Source: Horm Behav. 2017 Jul;93:99–108. doi: 10.1016/j.yhbeh.2017.05.011 (PMC5552616; doi:10.1016/j.yhbeh.2017.05.011)
Supplement: Supplementary Fig. 2 — Descriptive statistics for behavioral latencies. Light grey bars represent the Initial Latencies (neophobia response); medium grey bars represent the Reward Latencies; dark grey bars represent the Startle Latencies (risk-taking response). Values in parentheses indicate number of completed trials. There was a significant main effect of latency type (mean ± SD, sec: Initial Latencies: 347.8 ± 279.9; Reward Latencies: 143.5 ± 163.8; Startle Latencies: 283.3 ± 232.0; F2 = 22.82, p = 2 × 10− 6). There was no main effect of repeat number (F2 = 1.54, p = 0.233), suggesting that there was generally no habituation/sensitization effect of repeated measurements. There was, however, an interaction between latency type and repeat number (F4 = 3.08, p = 0.024), which was due to a reduction in the Initial Latencies between the first and second repeated trials (pairwise comparison: p = 0.01; no other pairwise comparison was significant), perhaps suggesting that Initial Latency measurements are sensitive to repeated measures wherein birds become habituated as the novelty of the platform declines. [file mmc2.pdf]

S2

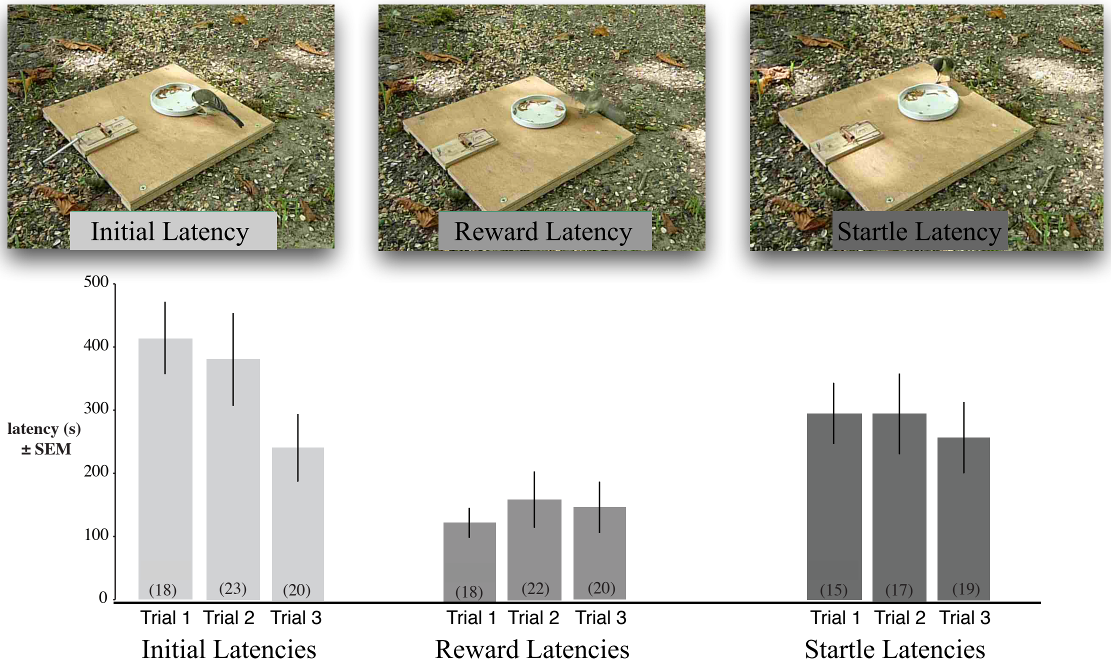

S2. Descriptive statistics for behavioral latencies. Light grey bars represent the Initial Latencies (neophobia response); medium grey bars represent the Reward Latencies; dark grey bars represent the Startle Latencies (risk-taking response). Values in parentheses indicate number of completed trials. There was a significant main effect of latency type (mean ± SD, sec: Initial Latencies: 347.8 ±

S2. Descriptive statistics for behavioral latencies. Light grey bars represent the Initial Latencies (neophobia response); medium grey bars represent the Reward Latencies; dark grey bars represent the Startle Latencies (risk-taking response). Values in parentheses indicate number of completed trials. There was a significant main effect of latency type (mean ± SD, sec: Initial Latencies: 347.8 ± 279.9; Reward Latencies: 143.5 ± 163.8; Startle Latencies: 283.3 ± 232.0; F_2=22.82, p=2 x 10^{-6}). There was no main effect of repeat number (F_2=1.54, p=0.233), suggesting that there was generally no habituation/sensitization effect of repeated measurements. There was, however, an interaction between latency type and repeat number (F_4=3.08, p=0.024), which was due to a reduction in the Initial Latencies between the first and second repeated trials (pairwise comparison: p=0.01; no other pairwise comparison was significant), perhaps suggesting that Initial Latency measurements are sensitive to repeated measures wherein birds become habituated as the novelty of the platform declines.
