# Supplementary material for: Risk-averse personalities have a systemically potentiated neuroendocrine stress axis: A multilevel experiment in Parus major
Source: Horm Behav. 2017 Jul;93:99–108. doi: 10.1016/j.yhbeh.2017.05.011 (PMC5552616; doi:10.1016/j.yhbeh.2017.05.011)
Supplement: Supplementary Fig. 3 — Repeated HPA assessments #1 (August 2012) and #2 (November 2012). Corticosterone concentrations were lower in August (main effect of season: F1,24 = 24.29, p < 5 × 10− 6) and there was a main effect of HPA component (F3,72 = 186.8, p = 1 × 10− 7). Lastly, there was an interaction effect between season and HPA component (F3,72 = 25.06, p < 1 × 10− 7), driven by weakened negative feedback (higher DexCORT) in November and a concomitant increase in adrenal sensitivity (higher ActhCORT). These results mirror the validation study (see Supplementary fig. 1). [file mmc3.pdf]

S3

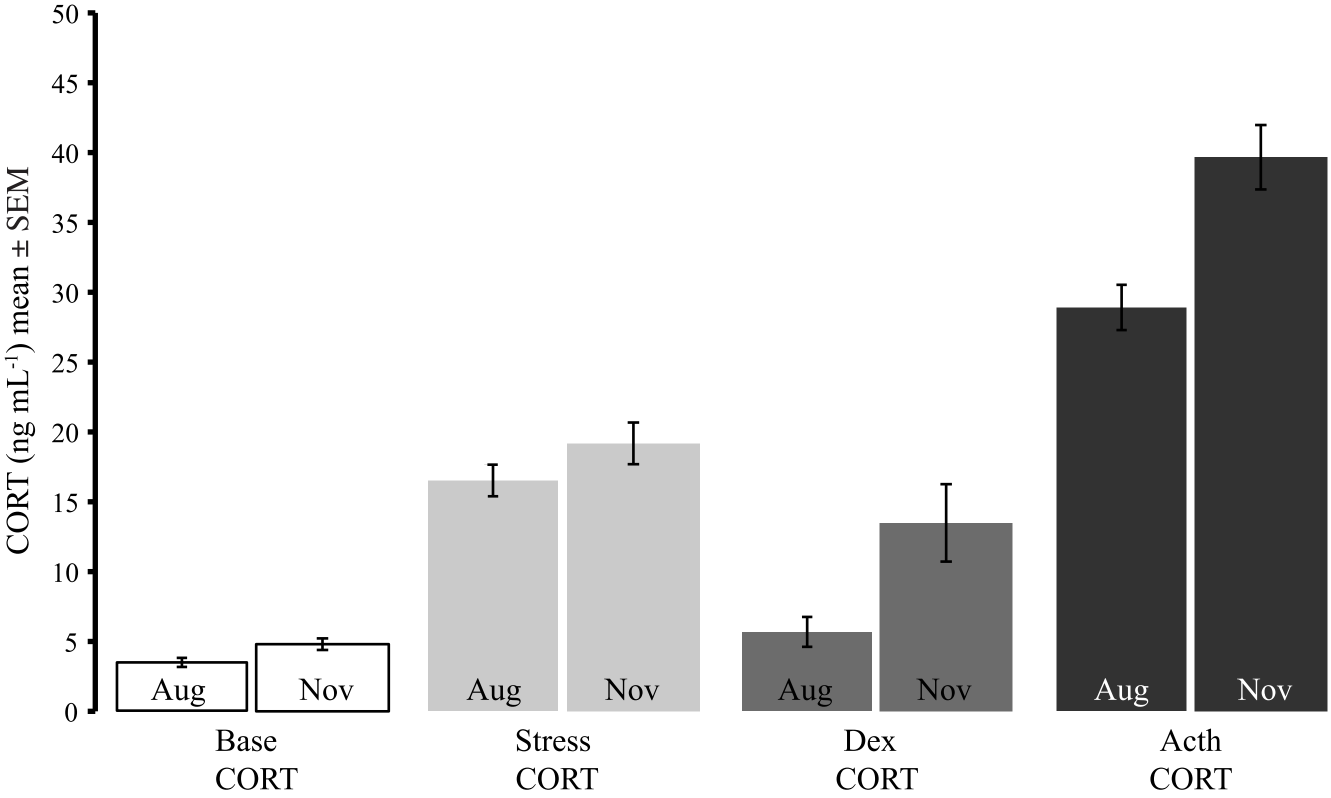

S3. Repeated HPA assessments #1 (August 2012) and #2 (November 2012). Corticosterone concentrations were lower in August (main effect of season: $F_{1,24}$=24.29, $p<5x10^{-6}$) and there was a main effect of HPA component ($F_{3,72}$=186.8, $p=1x10^{-7}$). Lastly, there was an interaction effect between season and HPA component ($F_{3,72}$=25.06, $p<1x10^{-7}$), driven by weakened negative feedback (higher DexCORT) in November and a concomitant increase in adrenal sensitivity (higher ActhCORT). These results mirror the validation study (see S1).
