# Supplementary material for: Risk-averse personalities have a systemically potentiated neuroendocrine stress axis: A multilevel experiment in Parus major
Source: Horm Behav. 2017 Jul;93:99–108. doi: 10.1016/j.yhbeh.2017.05.011 (PMC5552616; doi:10.1016/j.yhbeh.2017.05.011)
Supplement: Supplementary Table 4 — Adjusted repeatability estimates for the behavioral measures and the four HPA components. § Adjusted repeatabilities have SMI and fat score fitted as fixed effects (covariates) and individual as random effect for the R column and individual and nest of origin for the Nest ID column. †† Adjusted repeatabilities have SMI fitted as a fixed effect (covariate) and fat score fitted as a fixed effect (factor). [file mmc4.pdf]

S4

Trait		R	95CI	Nest ID	Nest ID 95CI
**Behaviour**					
Initial Latency	Adjusted[§]	0.34	0.11–0.62	-	-
Reward Latency	Adjusted[§]	0.55	0.24–0.75	-	-
Startle Latency	Adjusted[§]	0.53	0.22–0.77	-	-
**HPA**					
BaseCORT	Adjusted[§]	0.09	0.02–0.35	0.11	0.03–0.48
	Adjusted[††]	0.10	0.02–0.40	0.12	0.03–0.46
StressCORT	Adjusted[§]	0.12	0.03–0.44	0.12	0.03–0.42
DexCORT	Adjusted[§]	0.31	0.7–0.62	0.12	0.03–0.52
ActhCORT	Adjusted[§]	0.27	0.07–0.61	0.20	0.05–0.58

[§] Adjusted repeatabilities have SMI and fat score fitted as fixed effects (covariates) and individual as random effect for the R column and individual and nest of origin for the Nest ID column.
[††] Adjusted repeatabilities have SMI fitted as a fixed effect (covariate) and fat score fitted as a fixed effect (factor).
