# Supplementary material for: Risk-averse personalities have a systemically potentiated neuroendocrine stress axis: A multilevel experiment in Parus major
Source: Horm Behav. 2017 Jul;93:99–108. doi: 10.1016/j.yhbeh.2017.05.011 (PMC5552616; doi:10.1016/j.yhbeh.2017.05.011)
Supplement: Supplementary Fig. 5 — Graphical representations of variance and covariance components for the three behavioral traits (Initial Latency, Reward Latency, Startle Latency). All values are log10-transformed and plotted as standardized (z) scores and best fit lines are linear regressions. Plots a–c provide a partial (the first two of three repeated trials) illustration of the repeatability of each behavior. Positive correlations graphically indicate repeatable traits. See Table 1 for the repeatability estimates. Plots d–f illustrate the phenotypic correlation between Initial and Reward Latencies (i.e. the correlation between the traits during each sampling period). The positive correlations here indicate that there exists either a within- or an among-individual correlation (or both jointly) between these two traits. Plot g shows the correlation between the average (per bird) values of Initial and Reward Latencies across all three repeated trials. A positive correlation here is graphical evidence of an among-individual correlation (i.e. syndrome) between these two traits. Plot h depicts the correlation between the deviation from the average (per bird) values of Initial and Startle Latencies across all three repeated trials (i.e. each bird has three points on this plot). A positive correlation here is graphical evidence of a within-individual correlation (i.e. plasticity) between these two traits. Plots i–m illustrate the same relationships for Initial vs Startle Latencies. All graphs with linear regression lines demonstrated statistical signficance (see Table 1 for repeatability estimates and Table 3 for the within- and among-individual covariance estimates). See Baugh et al. (2014) for a complete description of these graphical methods. [file mmc5.pdf]

S5

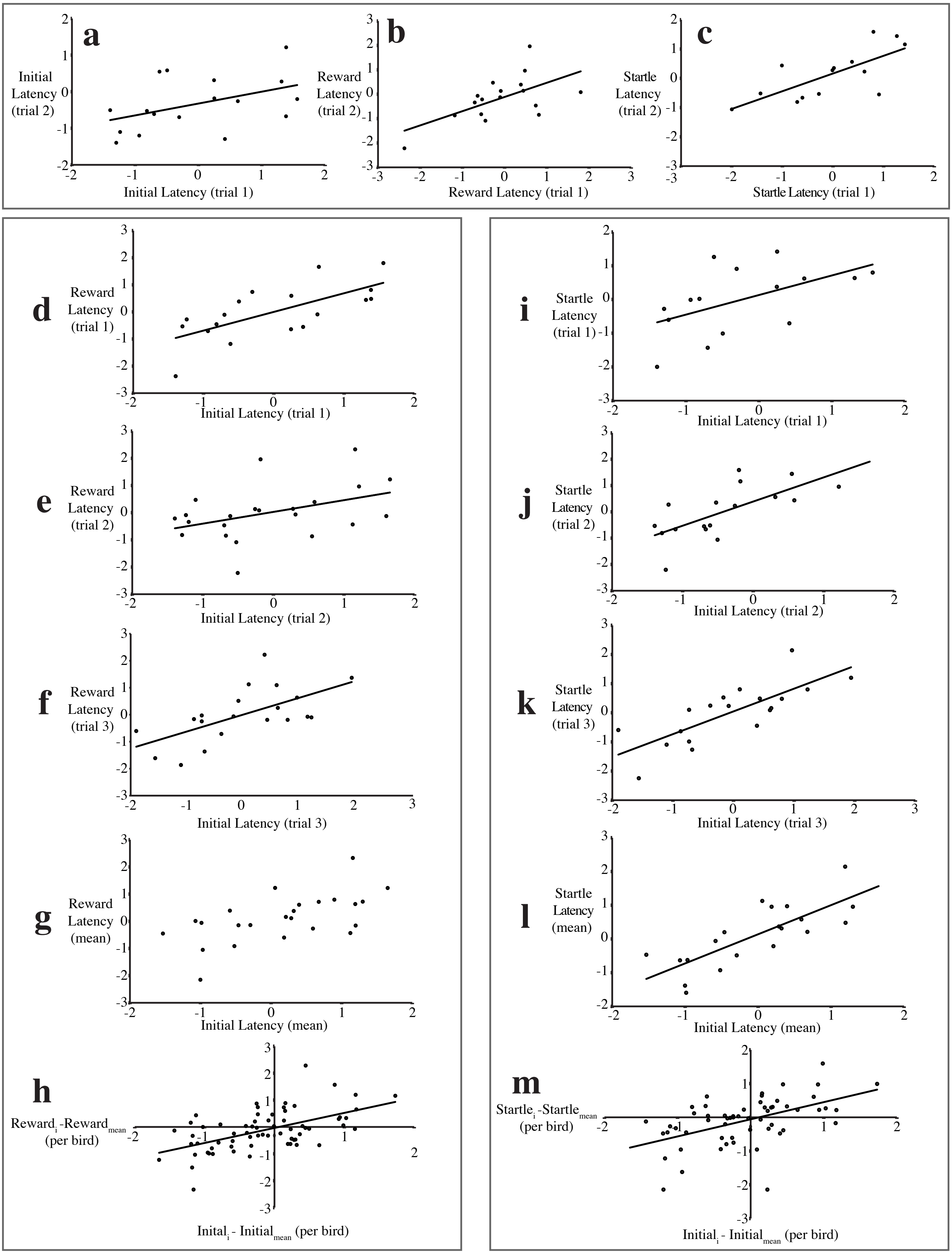

S5. Graphical representations of variance and covariance components for the three behavioural traits (Initial Latency, Reward Latency, Startle Latency). All values are $\log_{10}$-transformed and plotted as standardized (z) scores and best fit lines are linear regressions. Plots **a-c** provide a partial (the first two of three repeated trials) illustration of the repeatability of each behavior. Positive correlations graphically indicate repeatable traits. See Table 1 for the repeatability estimates. Plots **d-f** illustrate the phenotypic correlation between Initial and Reward Latencies (i.e. the correlation between the traits during each sampling period). The positive correlations here indicate that there exists either a within- or an among-individual correlation (or both jointly) between these two traits. Plot **g** shows the correlation between the average (per bird) values of Initial and Reward Latencies across all three repeated trials. A positve correlation here is graphical evidence of an among-individual correlation (i.e. syndrome) between these two traits. Plot **h** depicts the correlation between the deviation from the average (per bird) values of Initial and Startle Latencies across all three repeated trials (i.e. each bird has three points on this plot). A positve correlation here is graphical evidence of a within-individual correlation (i.e. plasticity) between these two traits. Plots **i-m** illustrate the same relationships for Initial vs Startle Latencies. All graphs with linear regression lines demonstrated stasitical signficance (see Table 1 for repeatability estimates and Table 3 for the within- and among-individual covariance estimates). See Baugh et al. (2014) for a complete description of these graphical methods.
