# Supplementary material for: Risk-averse personalities have a systemically potentiated neuroendocrine stress axis: A multilevel experiment in Parus major
Source: Horm Behav. 2017 Jul;93:99–108. doi: 10.1016/j.yhbeh.2017.05.011 (PMC5552616; doi:10.1016/j.yhbeh.2017.05.011)
Supplement: Supplementary Fig. 6 — Graphical representations of variance and covariance components for the four HPA components (BaseCORT, StressCORT, DexCORT, ActhCORT). All values are log10-transformed and plotted as standardized (z) scores and best fit lines are linear regressions. Plots a–d provide an illustration of the repeatability of each trait. Positive correlations graphically indicate repeatable traits. See Table 2 for the repeatability estimates. Plots e–f illustrate the phenotypic correlation between BaseCORT and StressCORT (i.e. the correlation between the traits during each sampling period). The positive correlations here indicate that there exists either a within- or an among-individual correlation (or both jointly) between these two traits. Plot g shows the correlation between the average (per bird) values of BaseCORT and StressCORT across both repeated HPA assessments. A positive correlation here is graphical evidence of an among-individual correlation (i.e. syndrome) between these two traits. Plot h depicts the correlation between the deviation from the average (per bird) values of BaseCORT and StressCORT across both repeated HPA assessments (i.e. each bird has two points on this plot). A positive correlation here is graphical evidence of a within-individual correlation (i.e. plasticity) between these two traits. See Table 3 for the within- and among-individual covariance estimates. Plots i–l illustrate the same relationships for StressCORT vs DexCORT, and plots m–p do so for DexCORT vs ActhCORT. All graphs with linear regression lines demonstrated statistical signficance (see Table 2 for repeatability estimates and Table 3 for the within- and among-individual covariance estimates). See Baugh et al. (2014) for a complete description of these graphical methods. [file mmc6.pdf]

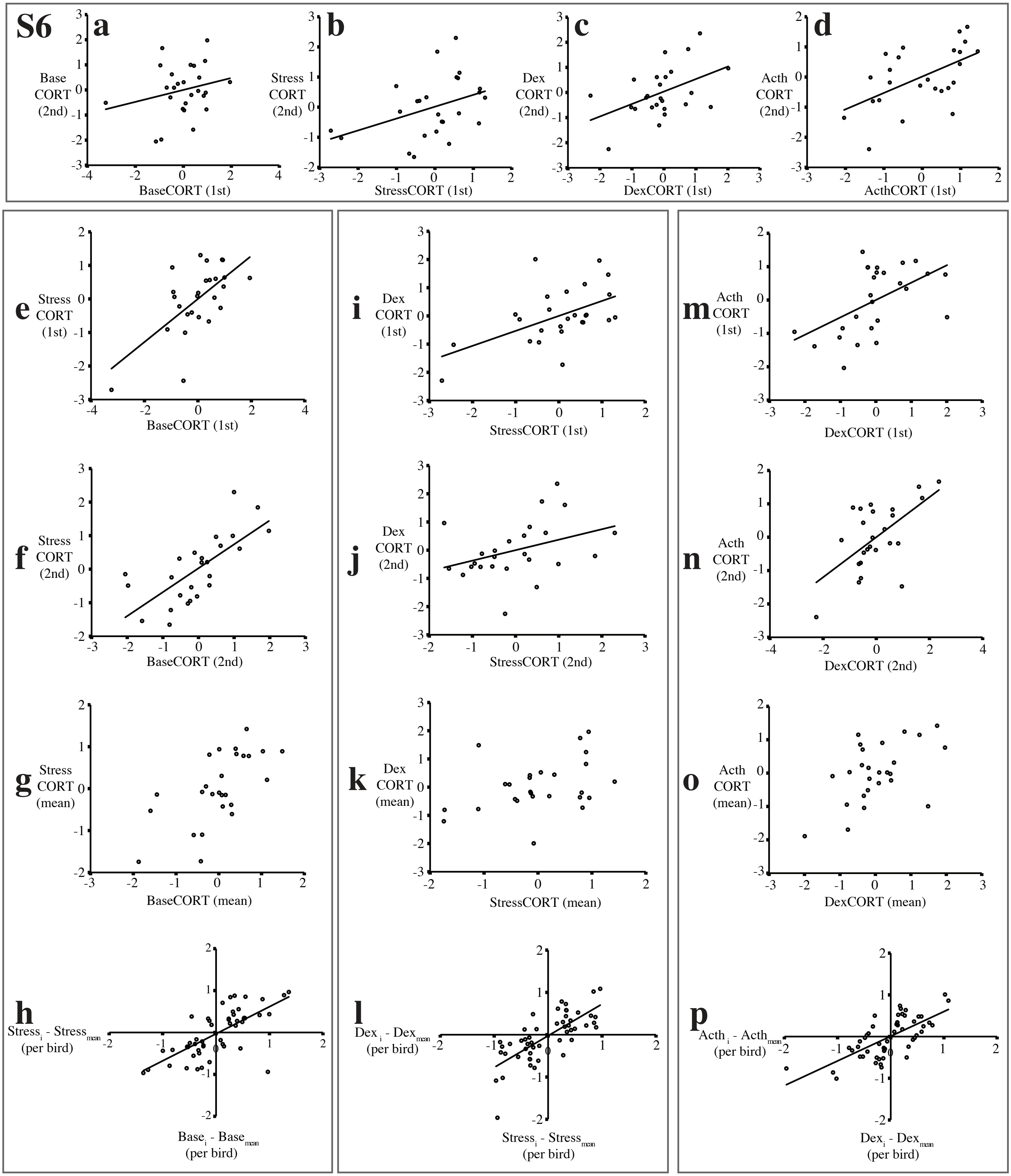

S6. Graphical representations of variance and covariance components for the four HPA components (BaseCORT, StressCORT, DexCORT, ActhCORT). All values are $\log_{10}$-transformed and plotted as standardized (z) scores and best fit lines are linear regressions. Plots **a-d** provide an illustration of the repeatability of each trait. Positive correlations graphically indicate repeatable traits. See Table 2 for the repeatability estimates. Plots **e-f** illustrate the phenotypic correlation between BaseCORT and StressCORT (i.e. the correlation between the traits during each sampling period). The positive correlations here indicate that there exists either a within- or an among-individual correlation (or both jointly) between these two traits. Plot **g** shows the correlation between the average (per bird) values of BaseCORT and StressCORT across both repeated HPA assessments. A positve correlation here is graphical evidence of an among-individual correlation (i.e. syndrome) between these two traits. Plot **h** depicts the correlation between the deviation from the average (per bird) values of BaseCORT and StressCORT across both repeated HPA assessments (i.e. each bird has two points on this plot). A positve correlation here is graphical evidence of a within-individual correlation (i.e. plasticity) between these two traits. See Table 3 for the within- and among-individual covariance estimates. Plots **i-l** illustrate the same relationships for StressCORT vs DexCORT, and plots **m-p** do so for DexCORT vs ActhCORT. All graphs with linear regression lines demonstrated stasitical signficance (see Table 2 for repeatability estimates and Table 3 for the within- and among-individual covariance estimates). See Baugh et al. (2014) for a complete description of these graphical methods.
