# Supplementary material for: Risk-averse personalities have a systemically potentiated neuroendocrine stress axis: A multilevel experiment in Parus major
Source: Horm Behav. 2017 Jul;93:99–108. doi: 10.1016/j.yhbeh.2017.05.011 (PMC5552616; doi:10.1016/j.yhbeh.2017.05.011)
Supplement: Supplementary Table 7 — a. General linear models predicting the second HPA assessment CORT secretion using log10 transformed MR and GR expression (in the PVN and HP) and log10 transformed CORT concentrations, with SMI as a covariate and fat score as a cofactor. Significant models (p < 0.05) are shown in white squares and non-significant models in grey squares. Bold text indicates the receptor expression was significant (p < 0.05) in predicting CORT concentrations in that model. Akaike Information Criteria corrected for small sample size (AICc; N = 25) are given for each model to evaluate fit, with smaller values indicating a better fit. Omnibus (“model”) statistics are reported in addition to statistics for the specific effect of receptor expression. Results were qualitatively identical when we applied hierarchical linear modeling techniques with NestID as a random factor. b. General linear models without fixed effects predicting the HPA2 (November samples) components using log10 transformed MR and GR expression (in the PVN and HP) and log10 transformed CORT concentrations. Akaike Information Criteria corrected for small sample size (AICc; N = 25) are given for each model to evaluate fit, with smaller values indicating a better fit. Significant models (p < 0.05) are shown in white squares and nonsignificant models in grey squares. Results were qualitatively identical when we applied standard least squares regression techniques. [file mmc7.pdf]

S7a. General linear models predicting the second HPA assessment CORT secretion using $\log_{10}$ transformed MR and GR expression (in the PVN and HP) and $\log_{10}$ transformed CORT concentrations, with SMI as a covariate and fat score as a cofactor. Significant models ($p<0.05$) are shown in white squares and non-significant models in gray squares. Bold text indicates the receptor expression was significant ($p<0.05$) in predicting CORT concentrations in that model. Akaike Information Criteria corrected for small sample size (AICc; N=25) are given for each model to evaluate fit, with smaller values indicating a better fit. Omnibus ("model") statistics are reported in addition to statistics for the specific effect of receptor expression. Results were qualitatively identical when we applied hierarchical linear modeling techniques with NestID as a random factor.

	GR in HP	GR in PVN	MR in HP	MR in PVN
Base CORT (trunk blood at sacrifice)	**χ^2_{model}=6.80,**<br>**p_{model}=0.34**<br>**β_{GRHP}= -1.24**<br>**χ^2_{GRHP}=4.54**<br>**p_{GRHP}=0.03***<br>**AICc=23.3**	**χ^2_{model}=6.26**<br>**p_{model}=0.39**<br>**β_{GRPVN} = -1.06**<br>**χ^2_{GRPVN}=4.02**<br>**p_{GRHP}=0.045***<br>**AICc=26.6**	χ^2_{model}=2.42<br>p_{model}=0.88<br>β_{MRHP}= -0.14<br>χ^2_{MRHP}=0.15<br>p_{MRHP}=0.70<br>AICc=27.7	χ^2_{model}=2.38<br>p_{model}=0.88<br>β_{MRPVN} = -0.15<br>χ^2_{MRPVN} =0.13<br>p_{MRPVN}=0.72<br>AICc=30.5
Base CORT	χ^2_{model}=10.3<br>p_{model}=0.11<br>β_{GRHP}= -0.54<br>χ^2_{GRHP}=2.10<br>p_{GRHP}=0.15<br>AICc=2.22	χ^2_{model}=10.3<br>p_{model}=0.11<br>β_{GRPVN} = -0.43<br>χ^2_{GRPVN} =1.82<br>p_{GRPVN}=0.18<br>AICc=5.35	**χ^2_{model}=13.0,**<br>**p_{model}=0.044***<br>**β_{MRHP}= -0.47,**<br>**χ^2_{MRHP} =4.75,**<br>**p_{MRHP}=0.029*,**<br>**AICc=-0.42**	χ^2_{model}=9.53<br>p_{model}=0.15<br>β_{MRPVN} = -0.25<br>χ^2_{MRPVN} =1.06<br>p_{MRPVN}=0.30<br>AICc=6.11
Stress CORT	**χ^2_{model}=14.5**<br>**p_{model}=0.025***<br>**β_{GRHP}= -0.56**<br>**χ^2_{GRHP}=3.95**<br>**p_{GRHP}=0.047***<br>**AICc= -12.58**	χ^2_{model}=9.87<br>p_{model}=0.13<br>β_{GRPVN} = 0.11<br>χ^2_{GRPVN} =0.21<br>p_{GRPVN}=0.65<br>AICc= -9.17	**χ^2_{model}=17.3,**<br>**p_{model}=0.008*,**<br>**β_{MRHP}= -0.42,**<br>**χ^2_{MRHP} =6.74,**<br>**p_{MRHP}=0.0094*,**<br>**AICc= -15.4**	χ^2_{model}=10.8<br>p_{model}=0.09<br>β_{MRPVN} = -0.19<br>χ^2_{MRPVN} =1.19<br>p_{MRPVN}=0.27<br>AICc= -10.15
Dex CORT	χ^2_{model}=6.19<br>p_{model}=0.40<br>β_{GRHP}= -1.08<br>χ^2_{GRHP}=2.15<br>p_{GRHP}=0.39<br>AICc=36.2	χ^2_{model}=4.10<br>p_{model}=0.66<br>β_{GRPVN} = 0.39<br>χ^2_{GRPVN} =0.34<br>p_{GRPVN}=0.56<br>AICc=39.4	χ^2_{model}=5.30<br>p_{model}=0.51<br>β_{MRHP}= -0.50<br>χ^2_{MRHP}=1.27<br>p_{MRHP}=0.26<br>AICc=37.1	χ^2_{model}=3.79<br>p_{model}=0.71<br>β_{MRPVN} = -0.08<br>χ^2_{MRPVN} =0.027<br>p_{MRPVN}=0.87<br>AICc=39.8
Acth CORT	χ^2_{model}= 19.5,<br>p_{model}=0.0034*<br>β_{GRHP}= -0.36<br>χ^2_{GRHP}=2.86<br>p_{GRHP}=0.09<br>AICc= -25.7	χ^2_{model}=14.9,<br>p_{model}=0.021*<br>β_{GRPVN} = -0.18<br>χ^2_{GRPVN} =0.85<br>p_{GRPVN}=0.36<br>AICc= -17.6	χ^2_{model}=17.5<br>p_{model}=0.0077*<br>β_{MRHP}= -0.12<br>χ^2_{MRHP} =0.82<br>p_{MRHP}=0.37<br>AICc= -23.6	χ^2_{model}=14.0<br>p_{model}=0.029*<br>β_{MRPVN} = 0.016<br>χ^2_{MRPVN} =0.012<br>p_{MRPVN}=0.91<br>AICc= -16.7

S7b. General linear models without fixed effects predicting the HPA_2 (November samples) components using $\log_{10}$ transformed MR and GR expression (in the PVN and HP) and $\log_{10}$ transformed CORT concentrations. Akaike Information Criteria corrected for small sample size (AICc; N=25) are given for each model to evaluate fit, with smaller values indicating a better fit. Significant models ($p<0.05$) are shown in white squares and non-significant models in gray squares. Results were qualitatively identical when we applied standard least squares regression techniques.

	**GR in HP**	**GR in PVN**	**MR in HP**	**MR in PVN**
Base CORT (trunk blood at sacrifice)	χ^2_{GRHP}= 4.52<br>p_{GRHP}=0.033*<br>AICc= 7.71	χ^2_{GRPVN}= 2.37<br>p_{GRPVN}=0.12<br>AICc= 11.5	χ^2_{MRHP}= 0.009<br>p_{MRHP}=0.92<br>AICc= 12.2	χ^2_{GRPVN}= 0.002<br>p_{GRPVN}=0.96<br>AICc= -13.8
Base CORT	χ^2_{GRHP}= 2.24<br>p_{GRHP}=0.13<br>AICc= -7.54	χ^2_{GRPVN}= 0.68<br>p_{GRPVN}=0.41<br>AICc= -4.06	χ^2_{MRHP}= 0.61<br>p_{MRHP}=0.43<br>AICc= -5.92	χ^2_{GRPVN}= 0.43<br>p_{GRPVN}=0.51<br>AICc= -3.82
Stress CORT	χ^2_{GRHP}= 2.75<br>p_{GRHP}=0.097<br>AICc= -18.7	χ^2_{GRPVN}= 0.11<br>p_{GRPVN}=0.74<br>AICc= -18.4	χ^2_{MRHP}= 0.61<br>p_{MRHP}=0.44<br>AICc= -16.6	χ^2_{GRPVN}= 0.01<br>p_{GRPVN}=0.92<br>AICc= -18.3
Dex CORT	χ^2_{GRHP}= 3.59<br>p_{GRHP}=0.058<br>AICc= 20.97	χ^2_{GRPVN}= 0.22<br>p_{GRPVN}=0.64<br>AICc= 24.3	χ^2_{MRHP}= 0.83<br>p_{MRHP}=0.36<br>AICc= 23.7	χ^2_{GRPVN}= 0.68<br>p_{GRPVN}=0.41<br>AICc= 23.8
Acth CORT	χ^2_{GRHP}= 3.05<br>p_{GRHP}=0.08<br>AICc= -27.1	χ^2_{GRPVN}= 0.34<br>p_{GRPVN}=0.55<br>AICc= -22.0	χ^2_{MRHP}= 0.23<br>p_{MRHP}=0.63<br>AICc= -24.3	χ^2_{GRPVN}= 0.14<br>p_{GRPVN}=0.70<br>AICc= -21.9
