# Supplementary material for: Risk-averse personalities have a systemically potentiated neuroendocrine stress axis: A multilevel experiment in Parus major
Source: Horm Behav. 2017 Jul;93:99–108. doi: 10.1016/j.yhbeh.2017.05.011 (PMC5552616; doi:10.1016/j.yhbeh.2017.05.011)
Supplement: Supplementary Table 8 — Component loadings and variance explained for items in the principal components analyses. All data were log10 transformed and z-standardized prior to PCA analysis. [file mmc8.pdf]

S8. Component loadings and variance explained for items in the principal components analyses. All data were $\log_{10}$ transformed and z-standardized prior to PCA analysis.

PCA	Variance Explained: PC1	Items	PC1 loadings	Communalities	N	KMO	Bartlett (χ^2, df, p-value)
**Behavioural Latencies**							
Initial, Reward, Startle	71.8%	Initial	0.899	0.807	14	0.666	11.8, 3, 0.008
		Reward	0.855	0.733	14		
		Startle	0.783	0.611	14		
Initial, Startle	78.2%	Initial	0.885	0.782	14	0.500	4.42, 1, 0.036
		Startle	0.885	0.782	14		
**HPA-CORT**							
Base, Stress, Dex, Acth	63.0%	Base	0.731	0.535	25		
		Stress	0.909	0.827	25	0.693	32.3, 6, 0.00001
		Dex	0.761	0.579	25		
		Acth	0.759	0.576	25		
Base, Stress	82.1%	Base	0.906	0.821	25	0.500	11.98, 1, 0.001
		Stress	0.906	0.821	25		
Dex, Acth	73.4%	Dex	0.857	0.734	25	0.500	5.58, 1, 0.018
		Acth	0.857	0.734	25		
**MR & GR**							
MR in HP & PVN	88.5%	HP	0.941	0.885	23	0.500	18.48, 1, 0.00001
		PVN	0.941	0.885	23		
GR in HP & PVN	69.3%	HP	0.833	0.693	23	0.500	3.31, 1, 0.06
		PVN	0.833	0.693	23		

Correlations among hormone and behavior principal components:

We used the correlation matrix to compute PC scores without factor rotation. KMO: Kaiser-Meyer-Olkin measure of sampling adequacy and Bartlett's test of sphericity are included. The first principal component (PC1) for all three behavioral measurements (*Initial Latency, Reward Latency, Startle Latency*) was positively and significantly correlated with PC1 for all four hormones (*BaseCORT, StressCORT, DexCORT, ActhCORT*): r=0.487, R^2=0.237, $F_{1,12}$=3.726, p=0.078; Spearman's rho=0.565, p=0.035, N=14. PC1 for two behaviors of particular interest that showed an among-individual correlation (*Initial Latency*, *Startle Latency*) was positively and significantly correlated with PC1 for all four hormones (*BaseCORT, StressCORT, DexCORT, ActhCORT*): r=0.652, R^2=0.425, $F_{1,12}$=8.861, p=0.012; Spearman's rho=0.600, p=0.023, N=14. PC1 for two behaviors of particular interest that showed an among-individual correlation (*Initial Latency*, *Startle Latency*) was positively and significantly correlated with PC1 for two hormones exhibiting trends for among-individual correlations (*BaseCORT*, *StressCORT*): r=0.608, R^2=0.370, $F_{1,12}$=7.051, p=0.021; Spearman's rho=0.613, p=0.020, N=14. PC1 for two behaviors of particular interest and ones that show an among-individual correlation (*Initial Latency, Startle Latency*) was positively and significantly correlated with PC1 for two hormones exhibiting trends for among-individual correlations (*DexCORT*, *ActhCORT*): r=0.648, R^2=0.420, $F_{1,12}$=8.690, p=0.012; Spearman's rho=0.653, p=0.011, N=14. Despite small sample sizes the principal component estimates appear to successfully reduce these variable sets to biologically meaningful components, suggesting that both the behavioral and hormonal traits are highly integrated.
