# Supplementary material for: Risk-averse personalities have a systemically potentiated neuroendocrine stress axis: A multilevel experiment in Parus major
Source: Horm Behav. 2017 Jul;93:99–108. doi: 10.1016/j.yhbeh.2017.05.011 (PMC5552616; doi:10.1016/j.yhbeh.2017.05.011)
Supplement: Supplementary Fig. 9 — Correlations between the natural stress response (StressCORT) and Initial Latencies (a) and negative feedback (DexCORT) and Startle Latencies (b) from HPA1 assessment and RTA1, respectively. Larger StressCORT values indicate a stronger stress response and larger DexCORT values indicate weaker negative feedback strength (i.e. higher CORT concentrations following dexamethasone challenge). Trials in which birds did not approach the platform within the 20 min criterion are included; exclusion of these birds do not qualitatively change these linear relationships. [file mmc9.pdf]

S9

a

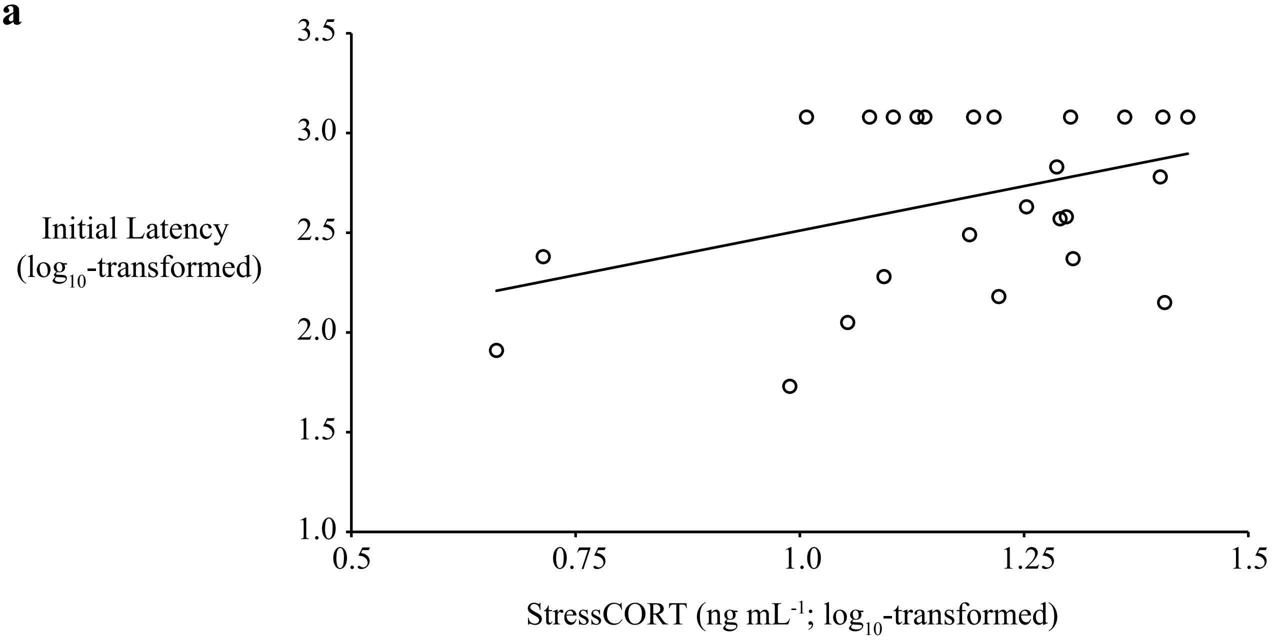

b

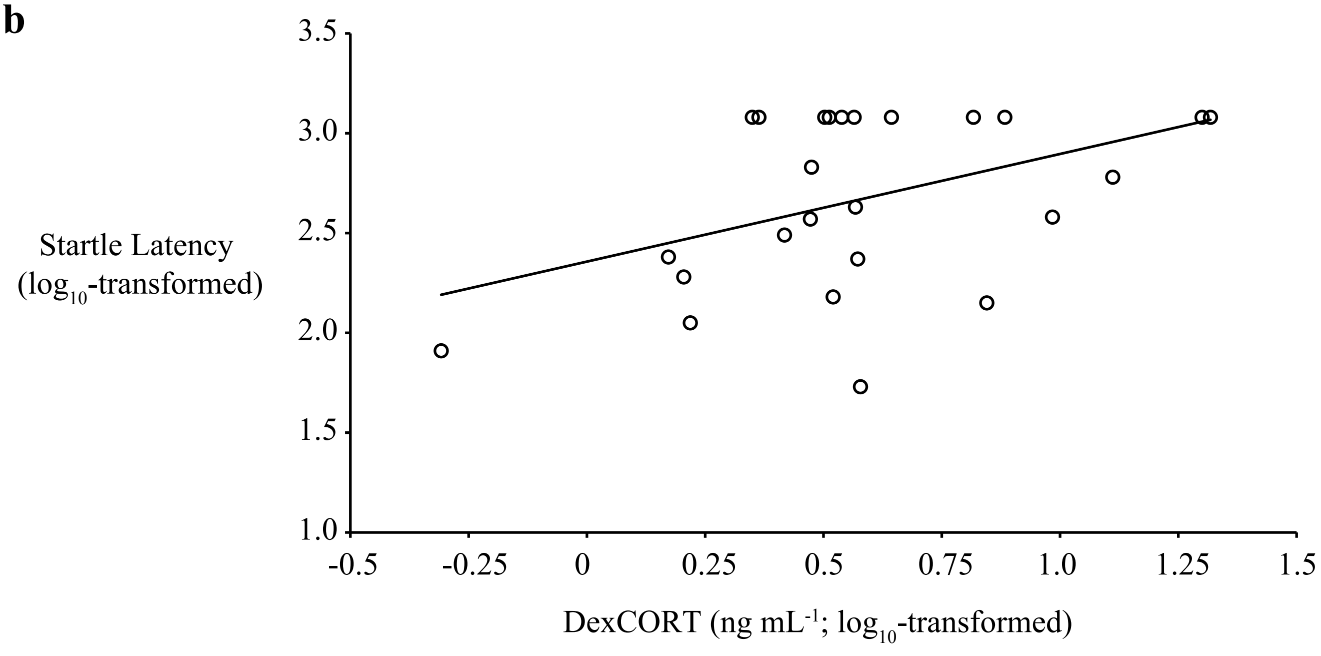

S9. Correlations between the natural stress response (StressCORT) and Initial Latencies (a) and negative feedback (DexCORT) and Startle Latencies (b) from HPA_1 assessment and RTA_1, respectively. Larger StressCORT values indicate a stronger stress response and larger DexCORT values indicate weaker negative feedback strength (i.e. higher CORT concentrations following dexamethasone challenge). Trials in which birds did not approach the platform within the 20 min criterion are included; exclusion of these birds do not qualitatively change these linear relationships.
